# Person-Centered Preventive Health Care: Gathering Stakeholder Input on Evidence and Implementation

**DOI:** 10.1016/j.focus.2025.100319

**Published:** 2025-01-31

**Authors:** Leila C. Kahwati, Hanan J. Aboumatar, Alison K. Banger, Sarah I. Bean, Laurie W. Hinnant, Daniel E. Jonas, Julia M. Kim, Jennifer S. Lin, Carrie D. Patnode, Meagan R. Pilar, Samantha I. Pitts, Shivani M. Reddy, Ritu Sharma, Christiane E. Voisin, Elizabeth M. Webber, Jodi Blake, Nora M. Mueller

**Affiliations:** 1 RTI International, Research Triangle Park, North Carolina; 2 Armstrong Institute for Patient Safety and Quality, Johns Hopkins University School of Medicine, Baltimore, Maryland; 3 Kaiser Permanente Center for Health Research, Portland, Oregon; 4 Division of General Internal Medicine, The Ohio State University College of Medicine, Columbus, Ohio; 5 Department of Pediatrics, Johns Hopkins University School of Medicine, Baltimore, Maryland; 6 Department of Medicine, Johns Hopkins University School of Medicine, Baltimore, Maryland; 7 Center for Evidence and Practice Improvement, Agency for Healthcare Research and Quality, Rockville, Maryland

**Keywords:** Patient-centered care, clinical preventive services, health equity, preventive service implementation

## Abstract

**Introduction:**

Clinical preventive services, such as screening tests, vaccinations, behavioral counseling, or preventive medication, are offered to most people on the basis of age, sex, health behaviors, or clinical risk factors, with goals of detecting early disease, preventing future disease, or mitigating the impact of unhealthy behaviors on future health. However, many people do not receive all the recommended services for which they are eligible.

**Methods:**

The Agency for Healthcare Research and Quality identified 4 topics for gathering stakeholder input on evidence and implementation for the equitable delivery of clinical preventive services. These included technology, innovative delivery models, public health linkages, and disparities. For each topic, the authors conducted an environmental scan to identify existing programs or interventions to promote the delivery of clinical preventive services, a technical expert panel meeting, and key informant interviews. The authors synthesized input from each topic's technical expert panel and key informant interviews and used inductive reasoning to identify themes. Within each overarching theme, the authors identified subthemes supported by specific statements, examples, and illustrative quotes.

**Results:**

A total of 90 individuals participated on stakeholder panels, technical expert panels, or key informant interviews; some individuals participated in multiple roles. Across the topics, the authors identified 3 overarching themes from synthesis of the technical expert panel and key informant interview comments across topics: (1) transitioning to holistic healthcare delivery and financing models, (2) including community and patient voice in healthcare system design, and (3) leveraging technology to improve clinical preventive services delivery.

**Conclusions:**

Promoting the equitable delivery of clinical preventive services requires improving access to primary care but also expanding efforts beyond clinical settings to encompass public health and community infrastructure and engagement. Experts recommended that person-centered preventive care should empower patients to make informed decisions about clinical preventive services on the basis of their values, risks, and preferences. This more individualized approach tailored to needs and context may reduce barriers to receipt of clinical preventive services.

## HIGHLIGHTS

• Realizing population benefits from clinical preventive services (CPSs) requires widespread and equitable access.
• Promoting the equitable delivery of CPSs requires improving access to primary care.
• Person-centered delivery of CPSs involves public health and community organizations.

## INTRODUCTION

Clinical preventive services (CPSs) recommended by the U.S. Preventive Services Task Force (USPSTF)^1^ or the Advisory Committee on Immunization Practices (ACIP),^2^ such as screening tests, vaccinations, behavioral counseling, or preventive medication, are offered to people on the basis of age, sex, health behaviors, or clinical risk factors with the goal of detecting early disease, preventing future disease, or mitigating the impact of unhealthy behaviors on future health. In the U.S., primary care clinicians provide most CPSs or refer people to specialty providers to obtain services when needed (e.g., colonoscopy for colorectal cancer screening). Evidence-based CPSs can reduce morbidity and mortality, helping people live longer, healthier lives, but many people do not receive all of the recommended services for which they are eligible, and some do not receive any.^1^^,^^3^, ^4^, ^5^, ^6^ The reasons for suboptimal receipt of CPSs are multifactorial and vary by type of service, individual factors, and system-level factors.^7^ Furthermore, existing disparities in health status, disease burden, and personal and community resources may contribute to gaps in equitable delivery of CPSs.^7^, ^8^, ^9^ Historically, racial and ethnic groups that have been marginalized, rural populations, sexual and gender minority populations, people with disabilities, people with serious mental illness, and individuals living in poverty experience a disproportionate burden of poor health outcomes and reduced access to care.^7^

Realizing the benefits from CPSs at a population level requires widespread and equitable opportunity for all to access them, including any downstream diagnostic evaluation or treatment. Although CPSs have the potential to improve the health of populations, it is critical that a person-centered approach is used. This means providing services in the context of a person's overall preferences and values and considering their individual risk factors, health status, and needs and what makes life meaningful for individuals outside of the healthcare setting.^10^ Beyond being the right thing to do,^11^^,^^12^ a person-centered approach is recommended to ensure equity, quality, responsiveness and participation, efficiency, and resilience in health care.^13^ However, little has been written about person-centered care in the context of CPSs.

Given the suboptimal and inequitable receipt of CPSs among adults, the Agency for Healthcare Research and Quality (AHRQ) commissioned the Person-centered Preventive Health Care project to gather evidence and stakeholder input across multiple topics. These topics were the use of technology in preventive health care, innovative preventive care delivery models, public health and primary care linkages, and health disparities in the context of CPSs. This paper outlines the findings of these discussions, with the intent to identify person-centered approaches to increase the equitable delivery of CPSs.

## METHODS

Detailed methods are provided in Appendix A (available online). Briefly, AHRQ identified 4 topic areas for this project (Table 1). For each topic, the authors assembled 3–4 people from their team to conduct an environmental scan, convene and facilitate a technical expert panel (TEP) meeting, and conduct key informant interviews (KIIs). During the 18-month project, the authors also virtually convened a 32-member stakeholder panel 3 times to provide guidance to the overall project, including suggestions for scoping the environmental scans, nominations for TEP members and KIIs, and feedback on draft findings. The stakeholder panel included representatives from healthcare systems, academia, public health agencies, nonprofit organizations, payers, federal agencies, and patient/consumer organizations.

**Table 1:** Topics Explored Within the Person-Centered Preventive Healthcare Project

Short topic title	Topic description
Technology	Identify how technology can be leveraged or developed to deliver equitable clinical preventive services, including personal health records and patient portals, mobile device applications, telehealth, and tools for facilitating shared decision making
Innovative delivery models	Identify emerging and innovative models, interventions, and/or programs that are implemented within healthcare organizations to ensure delivery of relevant preventive care in a way that incorporates patient values and preferences
Public health and community linkages	Identify how linkages between primary care and public health or community-based organizations can be further developed and leveraged to optimize the delivery of person-centered CPS
Disparities	Identify causes as well as identified person-centered strategies to mitigate health disparities related to CPS. The scope included but was not limited to strategies to improve the uptake of CPS across different populations

CPS, clinical preventive service.

The goal of the environmental scans was to provide the TEP members for each topic with an overview of the existing literature and example interventions, programs, or tools as well as identify salient questions to launch TEP discussions. For each topic, the authors developed guiding questions and searched 2 or 3 bibliographic databases from 2012 to the present, along with focused gray literature searches. Search terms included those related to CPSs, patient- and person-centered care, and topic-specific terms. Articles prioritized for the scan included systematic evidence reviews, primary research or quality improvement studies, and multistate demonstration projects to inform the understanding of CPS implementation. The authors synthesized the findings from the scan into a narrative highlighting evidence gaps. Additional methods details are provided in Appendix A (available online).

For each topic, the authors convened a TEP with at least 12 experts, including 2 patient/consumer representatives. The authors identified TEP members and key informants on the basis of stakeholder panel or AHRQ nominations or authors or contributors to information included in the environmental scans or through Internet searches for people working in the topic area. The authors used publicly available information on potential candidates to recruit TEP members across the U.S. representing different disciplines, genders, race and ethnicities, and organization types. The authors conducted each 2–3-hour TEP meeting virtually using Zoom, and each expert received an honorarium for their participation. The authors also used the XLeap virtual platform in most meetings to provide an additional forum for TEP members to share their thoughts. For each topic, the authors conducted 3–4 KIIs, either before or after the TEP meeting to gather additional information. The authors conducted KIIs through Zoom. Each TEP and KII was facilitated by a doctoral-level project team member with topic-specific content knowledge and at least 10 years of experience in such roles. Each facilitator used project-specific standard operating procedures developed using qualitative research best practices to facilitate the TEPs and the interviews. Given the diversity of topics, facilitators used a semistructured approach to the KIIs to allow for tailoring of questions to ensure that they met the unique needs of each topic area. The authors conducted the TEPs and KIIs between April and June 2023; the names and affiliations of persons who participated are provided in Appendix A (available online).

For each topic, the authors used information collected from the environmental scan and KIIs conducted prior to the TEP meeting to formulate discussion questions for the TEP meeting. However, the authors did not limit TEP discussions to these questions but allowed exploration of additional topics raised during the TEP discussion. After each TEP meeting and KII, members of each topic team met to identify themes on the basis of the TEP meeting transcript and KII notes and generated a written summary of the themes supported by example quotes.^14^ At the conclusion of all TEP and KIIs, the project team identified common themes present across the 4 topics with examples from each topic for each theme.^14^

## RESULTS

Ninety individuals participated in the stakeholder panel, 1 or more TEPs, or a KII (Table 2). For clarity, the authors will refer to stakeholder panel members, TEP members, and key informants as experts in the sections that follow. Across topics, the experts provided input that the authors categorized into 1 of 3 overarching themes, as follows: transitioning to holistic healthcare delivery and financing models, including community and patient voice in healthcare system design, and leveraging technology to improve preventive care delivery.

**Table 2:** Types of Organizations Represented on the Stakeholder Panel, TEPs, or KIIs

Organizational type	Number represented across stakeholder panel, TEPs, and KIIs
Federal agency	17
Health information technology	3
Healthcare system	16
Research/academia	14
Nonprofit organization/patient or consumer organization	11
Healthcare payer	3
State policy/public health agency	3
U.S. Preventive Services Task Force (current or former member)	6
Community Preventive Services Task Force (current or former member)	1
AHRQ Primary Care Learning Community	8
Other	8

AHRQ, Agency for Healthcare Research and Quality; KII, key informant interview; TEP, technical expert panel.

Within each overarching theme, the authors identified subthemes supported by specific examples and illustrative quotes. The authors acknowledge the interrelatedness of the overarching themes (and their corresponding subthemes) and some redundancy that readers may encounter.

### Transitioning to Holistic Healthcare Delivery and Financing Models

To increase the delivery of person-centered preventive care, experts proposed transforming the current healthcare system to a more holistic model. Experts discussed examples of holistic health care using 2 existing models: first, the Veterans Health Administration's Whole Health initiative,^15^ which uses a person-centered approach to health care and considers the impact that personal beliefs and values, experiences, and community can have on health and well-being, and second, Federally Qualified Health Centers, because these centers address social needs and provide comprehensive services (often including dental, vision, and mental health services) to people in underserved communities and include community representation in their governance. The authors identified 6 subthemes (Table 3).

**Table 3:** Subthemes Related to the Overarching Theme of Transitioning to Holistic Healthcare Delivery and Financing Models

Subtheme	Illustrative expert quotes
Expand perspective on preventive services	“We hear about screening for chronic diseases, and cancer. But we don't often talk about dental or periodontal issues and same thing about visual or auditory disorders; screening on a timely basis could reduce health disparities.” “We've got to start thinking about how we develop these individualized, personalized approaches so that we can address this for the broader population that will allow us to become much more equitable in our approach.”
Focus on person-centered delivery	“…Patient and community centered care with choice on how, where and with whom to increase access as well as trust... And shared decision making.” “I worry that the inherent message in quality measure benchmarking/performance goals is that the receipt of the service is the ‘right’ choice for the patient.”
Address social drivers of health and social needs	“If you ask a patient about their mental health or housing needs for example but have nothing to offer them or don't have time to discuss with them in depth you are fostering distrust.” “I have spent a lot of time in the HIV space and people are not interested in adherence or other health issues if they have no money and nowhere to sleep. [Basic needs are their] Priorities. ”
Invest in social and community infrastructure	“During the early days of covid, high numbers of the Latinx community were getting sick and being hospitalized but weren't getting tested or vaccinated. The [local health department] and [large academic medical center] set up sites in a large Latinx community to offer testing and vaccination and the impact was significant. the service went to the community.” “Current Diabetes Prevention Program payments do not account for the ongoing costs of community engagement, recruiting and enrolling participants, and the infrastructure needed to comply with payer and federal policies.”
Restructure fee-for service financing models	“Policies that reimburse for teams to deliver care are critical - there are not enough primary care providers to support the demand - the only way we can promote person-centered care and clinical preventive services is through team- based care. Without resources, the primary care provider cannot afford to hire support teams.” “If we're going to move to virtual and value-based care models, they need to be redesigned. We can't just continue hanging things off fee-for-service models … especially in preventive care and expect that we're going to suddenly get better outcomes.”
Identify opportunities for system redesign	“It isn't enough to refer our patients out to underfunded social service agencies. We must step up and invest in the systems that directly support the social needs that our patients have.” “It also is critically important to have a taxonomy that categorizes which services such as brief behavioral interventions in primary care such as tobacco cessation can be delivered on a telehealth platform. Many different allied health professionals can implement these.”

Experts recommended an expanded perspective on types of preventives services and a focus on person-centered delivery. They suggested a more inclusive view of CPSs beyond USPSTF or ACIP recommendations, for example, including dental, vision, and hearing screenings and maternal health care. They also suggested a life-course approach to prevention, which means taking a societal perspective to integrate prevention early in an individual's life, during life's transitions, and throughout one's lifetime, recognizing the interrelationships among people within current, past, and future generations.^16^^,^^17^ Although experts acknowledged the importance of evidence-based CPSs, many recommended more patient engagement and shared decision making in prioritizing and making decisions about receiving CPSs. They noted challenges associated with a one-size-fits-all approach to preventive care driven by CPS-related quality measures. Experts advocated for a care team that incorporates patient preferences, culture, and values into all aspects of health care, including CPSs.

Experts also identified the importance of addressing social drivers of health and social needs and investing in social services and community infrastructure. They described how social or economic factors—social determinants of health, such as transportation, education, income, and housing—can impact health behaviors and the ability to engage in care, including preventive care. Several experts suggested that although interventions with the potential to increase receipt of CPSs exist (in both research settings and real-world clinical settings), without broader, transformative change (i.e., interventions addressing social drivers of health), the ability to deliver CPSs and impact health outcomes is limited. They identified strategies at the policy, healthcare system, and provider levels (Appendix B, available online, and Appendix Table B1, available online) to address social needs. However, some cautioned that implementing some of these strategies without proper planning and resources could lead to distrust between patients and providers. For instance, screening patients for social needs without the availability of services to address needs identified would be detrimental for both patients and providers. Experts identified the need for greater investment in social services and community infrastructure, with the goal of enabling more team-based care and partnerships between healthcare systems and community organizations that have the resources and skills to address social needs outside the traditional realm of health care. They described various models for partnerships with community-based organizations (CBOs) and described the characteristics of successful partnerships (Appendix B, available online, and Appendix Table B2, available online).

Experts identified the need for restructuring fee-for-service models to enable more holistic models that include long-term, sustainable redesigns and community partnerships. Changes in healthcare delivery resulting from the coronavirus disease 2019 (COVID-19) pandemic have shifted public expectations and healthcare system capabilities for providing care, including increased care provision through telehealth and receipt of services such as lifestyle counseling and vaccinations outside of primary care, for example, through pharmacy clinics. Other system redesigns, such as the use of group-based care models, patient navigators, community health workers (CHWs), and Teaching Health Centers (i.e., embedding residency training in community health centers), may help transition the system toward a more holistic approach. However, experts advised that the use of these new models requires different financial incentives and alternative funding models to fee-for-service models to remedy the decades-long chronic underinvestment in primary care and public health and the lack of long-term funding for support of CBOs, which often rely on time-limited grants.

### Include Community and Patient Voice in Healthcare System Design

Experts offered comments on the importance of including the community and patient voice in the design of healthcare systems and models of care delivery, although their comments went beyond CPSs. They conveyed that integrating patient and community voice includes understanding a community and an individual's priorities in the context of their community and available resources and the cocreation of care delivery efforts. The authors identified 4 subthemes (Table 4).

**Table 4:** Subthemes Related to the Overarching Theme of Including Community and Patient Voice in Healthcare System Design

Subtheme	Illustrative expert quotes
Consider community priorities and context	“Understanding context, at multiple levels is critical to developing context-specific solutions that are relevant to individuals and communities.” “The NIH [National institutes of Health] CTSA[Clinical Translational Science Awards] program and NCI [National Cancer Institute] have been particularly useful for studying/developing the science of effective community engagement and trust building that leads to improved health equity.”
Cocreate care delivery and funding models	“Coming to community with preformed approaches and asking us for input is NOT the approach we are advocating for. Community MUST be equal partners from the outset.” “Similar to including the voice of the patient in intervention design, we should include the voice of policy makers and employers so that we can both directly get their input and potentially influence their thinking”
Design care delivery—where and who matters	“Building out the Community Health Worker [CHW] workforce. It [CHW workforce] is way too small to take on the task of integrating medical and social care as currently designed. Yet it has the capacity to offer real employment opportunities to many marginalized communities that are in need of work. There need to be more training programs/certification programs and ways to incorporate CHWs into the healthcare workforce as employees in order to do this work effectively and with the respect that these positions and populations deserve.” “I think we need to balance both the need for a relationship with a trusted person/provider, which is critical, but also not deny the health equity implications of having care and services convenient and nearby, particularly for marginalized communities. Imagine if we had not been able to get our COVID vaccines at our local pharmacy.”
Engage patients and communities in deimplementation decisions	“[There are racial disparities with prostate cancer incidence.] When screening for prostate cancer is called a “low-value” service, for whom is it low value?” “We have to bring the patient, family, and community voice into this work. We can't discuss patient and family-centered care strategies or policies if we aren't really engaging patients, families, and communities from the moment that we start to have these discussions… “

Experts shared that communities remain underrepresented in the health-related structures that exist today, including funding decisions, public health, primary care organizations, and healthcare systems. They shared that communities should be front and center of—if not leading and convening—partnerships and processes to inform health promotion models, including CPS delivery. In addition, although community involvement is desirable to understand community needs, it is equally important to consider when community priorities may not align with commonly accepted processes and practices for CPS delivery. Experts shared that trust is both critical and central to any effort to successfully engage and partner with communities and understand their priorities and important contextual considerations, particularly when working to improve health equity. They discussed how funding tied to a single condition, common for CPSs, is often based on funder priorities and that community representatives are engaged for their input on already designed interventions, which may or may not reflect the actual needs and priorities of the community.

Experts shared that although it may be easier to integrate feedback from community members into existing interventions and systems of care delivery and funding, a system redesign using a process of cocreation with community members, patients, families, and caregivers is needed. Experts identified the Practical Playbook series^18^ as a useful resource for establishing partnerships between primary care and public health, CBOs, and other sectors to increase access to CPSs. Critical to the process of cocreation is the identification of conveners—persons or groups that can take on the task of listening to the community, coordinating stakeholders, helping to shore up funding, facilitating data sharing, and providing infrastructure for smaller CBOs. Other key lessons learned from community engagement are provided in Appendix B (available online) and Appendix Table B3 (available online). Community engagement and trust were particularly relevant to healthcare system or provider efforts to curb the use of lower-value services (i.e., services with trivial to no effectiveness) because of misconceptions that equate such efforts with withholding necessary care, rationing, and confusion over medical reversals (the term that describes changes in recommendations that evolve on the basis of new research or information). Experts shared that mistrust or misunderstanding could negatively impact a patient's willingness to come in for services that they may benefit from, further widening disparities in access to high-value services.

When considering the design of care delivery, experts shared key considerations about where care is delivered and who is delivering it. This included the importance of an ongoing, trusted relationship with a primary care clinician or team but that affordable services based in the communities where individuals live may be a higher priority for many people, including those who may not have access to a primary care clinician. Experts discussed the value and importance of having a healthcare workforce that reflects the community it serves, from those providing frontline services to patients to those in executive leadership positions. They highlighted the importance of a sustained CHW labor force to enable closer linkages with the community. CHWs may go by different names, including doulas, peer support specialists, patient navigators, health coaches, lay workers, and promotoras, and serve in a variety of roles, such as conducting outreach to make connections with individuals and communities who are vulnerable, offering counseling or guidance on health behaviors, providing care coordination, advocating for individual and community health needs, and assisting people with enrollment in programs and benefits for which they are eligible. Experts emphasized the need for more CHW training and certification programs as well as the need for payers to consider CHWs as reimbursable services and not temporary positions often provided through external grants or funding. (In November 2023 [after TEPs were conducted], the Centers for Medicare & Medicaid Services finalized the 2024 Medicare Physician Fee Schedule, which among other things includes new coverage for services involving CHWs to address health-related social needs that impact care^19^).

Although experts emphasized the need for the community to contribute to the redesign of healthcare delivery systems, some were concerned that the resulting changes might impose an additional unintended burden on clinicians, especially primary care clinicians, who are reported to already be overwhelmed and under-resourced to meet both the complex needs of today's patient population and the administrative burden created by existing payment systems.^20^ The utilization of technology was often discussed as a key area for potential innovation; however, there were concerns about the burden that overlaying additional tools, resources, and systems could have on care delivery, especially if it results in more fragmented care.

### Leverage Technology to Improve Preventive Service Delivery

Experts offered details about structural issues that impact how technology could be used to deliver CPSs and equity considerations that must be addressed to improve care. The authors identified 4 subthemes (Table 5).

**Table 5:** Subthemes Related to the Leveraging Technology to Improve Preventive Service Delivery

Subtheme	Illustrative expert quotes
Provide technology to support the delivery of preventive services	“There is this idea of like semiautomatic personalization. We all ignore a spam email or a mass email, [but] it's a lot of work to handwrite or personalize something. How do we use technology to get us 80% of the way there and then personalize that last bit to be able to reach a patient where they are at?” “We need to think about who the stakeholders are and then how is this technology allowing us to empower those stakeholders to be able to deliver the care?”
Center equity in use of technology	“The more fancy and advanced we become in the use of technologic tools to deliver healthcare, the higher the risk of creating or exacerbating health inequities and disparities.” “And I don't know how you get paid to do digital health literacy. But if we're going to address health disparities, we need to also have an entire infrastructure set up for supporting a patient…”
Address technology implementation challenges	“The incorporation of these types of solutions into clinical practice is really quite hard, both from a workflow and a technical perspective.” “On the provider side the integration of structured data is so critically key and I kind of don't care if people giving me keep giving me read-only data, because if I can't write information back to the EHR you have actually added burden and effort to my work for no reimbursement. I now have to find the right place to enter the data and do it by hand, or I have to send a message to someone and ask them to do it for me, which is also more work for me and for them. So these things have to integrate, and they have to work in a more care management, friendly context.”
Address structural health information technology issues	“[Name of organization] has built several SMART on FHIR based tools and it is not easy at all to integrate those, even though we supposedly have a nationwide deployed version of a FHIR API that we should all be able to connect with. Nothing is plug and play. It takes money. It takes a bunch of experts and those experts need to be present in multiple places and all coordinating together if you want to get anything to actually work.” “The greatest challenge is not IT but capacity building. Some of that is with primary care, which is not funded nor accustomed to this side of the work, while local health departments are vastly underfunded and understaffed. CBOs likewise are thinly funded and staffed. The biggest need is building capacity—together.”

API, Application Programming Interface; CBO, community-based organization; EHR, electronic health record; FHIR, Fast Healthcare Interoperability Resources; IT, information technology; SMART, Substitutable Medical Applications, Reusable Technologies.

Technology could be used to support the delivery of more personalized preventive care. For example, technology could help individuals realize when a given recommendation might apply to them, assess their personal risks and benefits of the CPSs, or aid in the delivery of a CPS. Technology could also be used to improve coordination and communication among patients and care teams, particularly teams across multiple sites of care or organizations. However, clinical data standards and digital architecture are needed to understand and document individual preferences and values so that they are available to all care providers that a person might see.

The promising uses of technology mentioned included wearable health monitors and patient-generated data as well as artificial intelligence (AI) curation of existing data. Although technology has the potential to create efficiencies, experts identified a need for new roles, including someone within the health system to manage the trove of patient-generated data resulting from technological advancements. If patient-generated data are not standardized within the electronic health record or has no protocols for review, the healthcare team will not use these data. Experts also noted that regulations around AI are critical to protect against racial, gender, and other biases that may be amplified through the use of AI trained on biased data.

Current technology tools are not created with the needs of all patients in mind and may be inaccessible to people with limited English proficiency, lower health and/or digital literacy, limited access to technology, or communication challenges. Experts suggested that many patients may need a digital health navigator, someone who can provide guidance on how to use and engage with their health care through technology. They also noted that older adults and persons with disabilities may have special needs related to the use of technology that should be considered to ensure equity and maximize utility. Furthermore, some tools may not be appropriate for a person's cultural context or may be insensitive to a person's social needs, resulting in more harm than benefit. Experts emphasized that the use of technology could worsen disparities if the needs of diverse populations are not considered in the technology's design and implementation or if there is an overreliance on electronic means to communicate with patients. Similarly, not all patients feel comfortable with extensive use of technology to interact with their healthcare providers; the system must collect and act on patient preferences to best meet their needs.

Finally, experts emphasized that for technology to deliver on its promise, it needs to be effectively integrated into patient care so that it supports care delivery rather than distracting from it. A lack of effective technology integration could create an additional burden for providers and care teams rather than alleviating time and resource pressures. They gave examples of how 2 types of technology with the potential to support primary care—mobile applications and electronic health records—face several implementation challenges that may hinder progress. Furthermore, experts identified many health information technology infrastructure and data needs that the system must address to improve CPS delivery, including data governance, interoperability and data integration across healthcare systems and CBOs, and alignment of payments involving health information technology with desired clinician behaviors and outcomes.

## DISCUSSION

The AHRQ sponsored this project to collect information from experts about the person-centered, equitable delivery of CPSs through the lenses of 4 different topics: technology, innovative delivery models, public health linkages, and health disparities. The authors heard common sentiments from experts across these topics that the authors synthesized into 3 overarching themes. Although the authors planned each TEP and related KIIs to focus specifically on the delivery of CPSs, experts suggested that preventive care should encompass services beyond those defined by USPSTF and ACIP recommendations. They also contended that challenges with the equitable and person-centered provision of CPSs reflect larger, pervasive challenges within primary care, public health, and social supports within communities.

Experts emphasized community and patient voice in the design of holistic healthcare models to improve CPS delivery, but such models may not be successful or sustainable in the absence of an infrastructure that supports social needs for all. To achieve health equity, a holistic approach needs to be applied within health care and across the care continuum and sectors of society to address the social drivers of inequities. This is aligned with the concept of proportionate universalism: to reduce inequalities in health and well-being, universal services must be provided at a scale and intensity proportionate to the degree of need.^21^ To paraphrase one expert's comments, “engaging in preventive care is a luxury for many people it only happens when other basic needs are met and if it doesn't require undue hardship.” In late fall 2023, the U.S*. Playbook to Address Social Determinants of Health* was released and offers actions for federal agencies along 3 pillars: (1) expansion of data gathering and sharing, (2) support for flexible social needs funding, and (3) support for backbone organizations.^22^ This study's experts’ discussions were very much aligned with these pillars.

A tension between maximizing population health through the delivery of all recommended CPSs to all adults and person-centered care was a theme observed in expert discussions. Person-centered approaches challenge current quality measurement paradigms that incentivize the provision of some CPSs (typically, the ones easiest to measure) without consideration for whether those services are aligned with an individual's values and preferences. Holistic healthcare delivery and financing models that leverage the use of technology and paraprofessionals within health systems, communities, or both may ensure that all persons are offered the information that they need to make decisions about receiving CPSs, along with convenient access and coverage for those services. An analysis examining primary care visits over the period 2001–2019 found the greatest increases in preventive service delivery among Medicare beneficiaries in the years after the passage of the Affordable Care Act (2010), which authorized annual wellness visits for Medicare beneficiaries, demonstrating the significant impact that changes in reimbursement policy can have on CPS delivery.^23^

Given the demands on healthcare providers and a pressing need to treat acute conditions and manage chronic diseases, alternative CPS delivery settings (e.g., pharmacies), providers (e.g., CHWs), and technology (e.g., mobile health applications, telehealth) may offer opportunities to bridge existing gaps in delivery as well as bolster person-centered preventive care by providing CPSs at the right time and place of need. Although providers and systems should continue to strengthen their capacity to deliver CPSs to individuals in the context of an ongoing, longitudinal relationship, an expanded view of delivery settings and community collaboration, in tandem with social and ecologic drivers of CPS receipt, may offer solutions.

This study's experts’ discussions align with the National Academies of Sciences, Engineering, and Medicine (NASEM) report on Implementing High-Quality Primary Care.^10^ For example, NASEM calls for a variety of settings and modalities for care delivery and a stronger community orientation for providing primary care, similar to many experts’ suggestions for broadening the types of settings and staff that can provide CPSs. The need to leverage technology solutions to make CPSs more accessible and convenient to more persons is consistent with NASEM's identification of digital health as a facilitator for high-quality primary care.^10^ Several NASEM implementation objectives are highly relevant to the delivery of CPSs, including paying for primary care teams to care for people rather than paying doctors to deliver services; ensuring that high-quality care is available to every individual and family in every community; and designing information technology that serves the patient, family, and care team.^10^

### Limitations

The authors focused the environmental scans on U.S. settings, and they were not comprehensive systematic reviews. Published literature may be biased toward work that receives dedicated research funding, uses specific study designs, has positive findings, or focuses on disease-specific outcomes. By design, the authors limited TEPs for each topic to 12–15 people, and the authors conducted an average of 4 KIIs per topic. The authors strove to elicit the widest spectrum of input from experts, and the comments expressed were not necessarily consensus views. Although the authors aimed for a diverse panel, the experts the authors engaged likely did not fully represent the diversity of perspectives in the U.S. and, thus, may reflect an unrepresentative sample. The authors did not conduct standardized KII training or a formal qualitative data analysis of TEP meeting transcripts and KII notes (e.g., independent coding of data) and the team's beliefs and preferences may have unconsciously influenced the findings.

## CONCLUSIONS

Although the authors identified some interventions aimed at increasing person-centered delivery of CPSs, experts called for more holistic approaches to address health disparities, including addressing social drivers of health. They suggested a transition to holistic models of healthcare delivery and financing that incorporate community and patient voices in system design and that leverage technology-based solutions. Equity requires expansion beyond clinical settings to encompass public health and community infrastructure and engagement. Experts recommended that person-centered preventive care should enable individuals to make informed choices about CPSs on the basis of their values, risks, and preferences and support them in receiving chosen services within the context of their community. This approach tailored to individual needs and community context may reduce barriers to CPSs across diverse populations.

## Appendix Supplementary materials

Image, application 1

## Footnotes

Supplementary material associated with this article can be found in the online version at doi:10.1016/j.focus.2025.100319.
