# Supplementary material for: Person-Centered Preventive Health Care: Gathering Stakeholder Input on Evidence and Implementation
Source: AJPM Focus. 2025 Jan 31;4(2):100319. doi: 10.1016/j.focus.2025.100319 (PMC11880699; doi:10.1016/j.focus.2025.100319)
Supplement: Supplementary file 1 [file mmc1.docx]

# Online Appendix A. Detailed Methods

In this appendix, we provide additional details regarding the methods we used to convene the Stakeholder Panel, conduct four environmental scans, convene four technical expert panels (TEPs), and engage with key informants. The methods described in this appendix are overarching; please see the topic-specific sections of Appendix A for methods specific to each topic. This activities described were not considered research and were not required to undergo research ethics review.

Stakeholder Panel

The role of the Stakeholder Panel was to provide guidance to our team throughout the course of the 18-month project. This included:

- Providing overall project guidance
- Responding to questions regarding the scope of the environmental scans
- Suggesting individuals who can serve as key informants for the various topics
- Serving as a resource when recruiting and convening the TEPs
- Serving as TEP members, when appropriate and feasible
- Reviewing and providing thoughtful feedback on the final report by a subset of Stakeholder Panel members

Stakeholder Panel Recruitment

We identified 138 potential Stakeholder Panel members from a core set of organizational types (Table A-1). We considered factors such as role, organization type, clinical or specialty area, gender, geography, and self-identified racial or ethnic minority when compiling the list of potential TEP candidates. After AHRQ approved the list of candidates, we recruited potential members via email over several weeks in waves until 30 people agreed to participate; the list of Stakeholder Panel members is provided in Table A-2. We offered Stakeholder Panel members a $400 honorarium for their participation on the panel.^[[1]](#footnote-1)^

Table A-1. Types of Organizations Represented on the Stakeholder Panel

| Organizational Type | Number Represented on Panel |
| --- | --- |
| Federal agency | 7 |
| Health information technology | 2 |
| Healthcare system | 5 |
| Research/academia | 0* |
| Nonprofit organization/patient or consumer organization | 2 |
| Healthcare payer | 2 |
| State policy/public health agency | 2 |
| United State Preventive Services Task Force  (current or former member) | 2 |
| Community Preventive Services Task Force  (current or former member) | 1 |
| AHRQ Primary Care Learning Community | 7 |

* Individuals selected often represented multiple types of organizations; we identified several people who represented both research and academia along with another organizational type. Therefore, we did not recruit anyone solely from research/academia.

Abbreviations: AHRQ, Agency for Healthcare Research and Quality.

Table A-2. Stakeholder Panel Participants

| Name | Organization | Role | Type of Organization |
| --- | --- | --- | --- |
| Chethan Bachireddy, MD, MSc, FACP, AAHIVS | Harris Health | Chief Health Officer | Healthcare system |
| Nina Birnbaum, MD | Blue Cross Blue Shield Association of California | Medical Director, Health Transformation Acceleration | Payers |
| Maureen Boardman, MSN, FNP-C, FAANP | Little Rivers Health Care; Dartmouth Geisel School of Medicine | Director of Clinical Quality; Clinical Assistant Professor of Community and Family Medicine | AHRQ Primary Care Learning Community |
| Stacie Carney, MD | Oregon Community Health Information Network (OCHIN)—nonprofit innovation center providing health IT support services | Chief Medical Information Officer | Health information technology |
| Alison Cuellar, PhD | George Mason University | Vice Chair; Professor | Community Preventive Services Task Force (current/former member) |
| David Dietz, EdD, MSW, MHSA | Division of Healthcare Delivery, Innovation Center, Centers for Medicare & Medicaid Services | Director | Federal agency |
| Leslie Doroski McDowell, DNP, ANP-BC, RN | Wake Forest University/Northwest Area Health Education Center | Quality Improvement/Curriculum Development | AHRQ Primary Care Learning Community |
| Shannon Dowler, MD, FAAFP, CRE | State of North Carolina Department of Health and Human Services; Medicaid | Assistant Secretary for Health Access; Chief Medical Officer NC Medicaid | Payers |
| Stacy Garrett-Ray, MD, MPH, MBA | Ascension | Senior Vice President and Chief Community Impact Officer | Healthcare system |
| Howard Haft, MD, MMM, CPE, FACPE | American Heart Association Ambulatory Quality Committee, formerly with the Maryland Department of Health | Senior Advisor | State policy/public health agency |
| R. Scott Hammond, MD, AAFP | Colorado Center for Primary Care Innovation | Board President and Co-Founder | AHRQ Primary Care Learning Community |
| Tom Keane, MD, MBA (1st meeting)  David Hunt, MD, FACS (2nd meeting) | Office of the National Coordinator for Health Information Technology, Department of Health and Human Services | Senior Advisor (TK)  Medical Director (DH) | Federal agency |
| Jane Kim, MD, MPH | Veterans Health Administration, National Center for Health Promotion and Disease Prevention | Executive Director of Preventive Medicine | Federal agency |
| Alex Krist, MD, MPH | Virginia Commonwealth University | Professor and Co-Director, Ambulatory Care Outcomes | USPSTF (current/former member) |
| Nivedita Mohanty, MD, MS | Alliance Chicago | Chief Research Officer | AHRQ Primary Care Learning Community |
| David M. Murray, PhD (1st meeting)  Robert McNellis, MPH, PA (2nd meeting) | National Institutes of Health, Office of Disease Prevention | Associate Director for Prevention (DM)  Senior Advisor (RM) | Federal agency |
| Shilpa Patel, PhD | Center for Health Care Strategies | Associate Director for Health Equity | State policy/public health agency |
| Deborah Porterfield, MD, MPH | Formerly, Department of Family Medicine, University of North Carolina-Chapel Hill and North Carolina Department of Health and Human Services; currently, Office of the Assistant Secretary for Planning and Evaluation | Associate Professor; Medical Consultant; Medical Officer | Research/academia; State policy/public health agency; Federal agency |
| Paul Reed, MD, RADML U.S. Public Health Service | Office of Disease Prevention and Health Promotion, U.S. Department of Health and Human Services | Deputy Assistant Secretary for Health | Federal agency |
| Matthew Sakumoto, MD | Sutter Health | Chief Medical Information Officer, Virtual-First Primary Care Physician | AHRQ Primary Care Learning Community |
| Julie Schilz, BSN, MBA | Primary Care Development Corporation | Senior Director | AHRQ Primary Care Learning Community |
| Louise C. Walter, MD | University of California San Francisco | Professor and Chief of Division of Geriatrics | AHRQ Primary Care Learning Community |
| Scott Young, MD | Kaiser Permanent Care Management Institute | Executive Director | Healthcare system |
| Judy Zerzan-Thul, MD, MPH | Washington Health Care Authority | Chief Medical Officer | Healthcare system |
| Anonymous | - | - | 1 from Healthcare payer  2 from Health information technology organization  2 from Federal agencies  1 from Nonprofit organization  1 from USPSTF (current/former member) |

Abbreviations: USPSTF, U.S. Preventive Services Task Force; AHRQ, Agency for Healthcare Research and Quality, U.S., United States.

Stakeholder Panel Meetings

Prior to each Stakeholder Panel meeting, we worked with AHRQ to develop a detailed agenda, meeting pre-reads, and a presentation slide deck. The first Stakeholder Panel meeting was held in September 2022, the second meeting was held in June 2023, and the third meeting was held in Spring 2024. We conducted each meeting virtually using Zoom and each meeting lasted 2 hours. We offered Stakeholder Panel members a mechanism for providing additional feedback or suggestions for TEP members or key informants via an online form following each meeting. We developed meeting summaries from each Stakeholder Panel meeting**.** To provide updates between meetings, we developed and distributed a quarterly newsletter with brief project updates. Finally, we invited three Stakeholder Panel members and one patient/consumer representative that served as a TEP member to review and provide feedback on a draft version of the project’s final report.

Environmental Scan Overarching Methods

The goal of the environmental scan for each topic was to provide TEP members with a broad overview of the existing literature and other relevant background information. Each scan was conducted over approximately 12 weeks by a team consisting of a scan lead, an information specialist, one or more research analysts, one or more topic-specific experts, an implementation science expert, and editors.

Scoping of the Scans

Each scan began with the development of the topic scope and guiding questions to focus the search strategy. We sought input from the Stakeholder Panel and AHRQ staff, and for some topics, we conducted one or more key informant interviews to provide input to assist with scoping.

Searches and Article Selection

For each scan, we conducted bibliographic database searches (in 2 or 3 databases) along with grey literature and website searches. Search terms included those related to clinical preventive services and person-centered care, as well as topic-specific terms. Searches were limited to English language materials only and to studies published after 2010 (the passage of the Affordable Care Act, which included coverage for certain clinical preventive services). For some topics, the search dates were further limited. With some topic-specific exceptions, searches and article selection were limited to U.S. settings, but information from settings outside of the United States was sometimes included if it appeared in the search and was considered highly relevant.

We used a single person to screen titles and abstracts from our searches for relevant articles and information, with team discussions when needed. Full-text articles for relevant titles and abstracts were retrieved and reviewed. We prioritized the following article types for selection: systematic evidence reviews, narrative reviews and commentaries that placed evidence of effectiveness into a historical and present-day real-world context, as well as quality improvement studies and multistate demonstration projects to inform an understanding of implementation barriers and facilitators.

Information Gathering and Synthesis

From the selected articles and data sources, we gathered information relevant to each scan’s guiding questions and synthesized the major findings into themes using narrative and tabular formats with specific examples wherever possible. We also highlighted evidence gaps. The environmental scan team then worked closely with the team leading the TEP to develop a discussion guide for the panel.

Technical Expert Panel Overarching Methods

We recruited participants and facilitated a TEP meeting for each of the four topics included in this project.

Technical Expert Panel Recruitment

We recruited at least 12 experts, including 2 patient representatives for each TEP. We developed an initial list of about 30 potential TEP candidates for each topic based on recommendations from AHRQ, the Stakeholder Panel, information from the topic’s environmental scan, and our own knowledge about experts in the topic area. We considered factors such as role, organization type, clinical or specialty area, gender, geography, and self-identified racial or ethnic minority when compiling the list of potential TEP candidates. After AHRQ approved the list of candidates, we recruited potential members via email over several weeks in waves. We offered participants a $400 honorarium for their participation on the TEP.^[[2]](#footnote-2)^ A list of people who participated on each TEP is in the topic specific sections of this appendix**.**

Technical Expert Panel Meetings

Prior to each TEP meeting, we worked with AHRQ to develop a detailed agenda, meeting pre-reads, and presentation slide deck. We conducted each meeting virtually using Zoom and each meeting lasted 2 to 3 hours. Each TEP meeting was 2 to 3 hours long and was conducted virtually using Zoom. Most of the meetings also utilized the XLeap virtual meeting platform, which allows meeting participants to respond to important discussion questions during and after the meeting. It also allows for a virtual dialogue between participants, who they can view and respond to what other members share in real time. This tool was used to ensure that all TEP members had an opportunity to share their thoughts and fully participate in the meeting. We recorded all TEP meetings via the Zoom platform and we developed meeting summaries based on the Zoom recording, Zoom chat transcript, and XLeap contributions (if the platform was used) to summarize the TEP discussion.

Key Informant Interviews

For each topic, we identified between three and four potential key informants. In some cases, key informants had originally been recruited for the TEP but had scheduling conflicts that precluded their participation on the panel. In other cases, key informants were consulted prior to the TEP meeting to inform the topic scope or scan. However, most commonly, key informants were interviewed after the TEP meeting to obtain additional information or details around an issue or specific example that surfaced at the TEP meeting. After AHRQ approved the list of key informant candidates, we invited potential key informants via email. We conducted key informant interviews (KIIs) via Zoom and each interview lasted no longer than 60 minutes. We developed a tailored, semi-structured interview guide that consisted of four to six questions with additional probes. Each KII was recorded, and key findings were added as an addendum to each TEP Meeting Summary at the completion of all KIIs.

Topic-Specific Methods

Technology: Environmental Scan Data Sources and Searches

A search strategy (**Table A-3**) was developed in consultation between the evidence scan lead and an information specialist by building upon a 2022 scoping review by Willis and colleagues^24^ that focused on digital health interventions in primary care settings and by examining the indexing of known relevant articles. The information specialist searched PubMed from the years 2020 forward. Grey literature was identified through additional supplemental searches of PubMed and Google Scholar, HealthIT.gov, Agency for Healthcare Research and Quality’s (AHRQ’s) Digital Healthcare Research Program and Evidence-based Practice Center reports, and reference lists from recent review articles and editorials. All citations were managed and deduplicated using EndNote 20 (Clarivate Analytics).

**Table A-3**. Search Strategy for the Technology Topic Area

| Search  Number | PubMed Query | Results |
| --- | --- | --- |
| 1 | "primary health care"[MeSH] OR "Physicians, Primary Care"[MeSH] OR "primary care"[tiab] OR "Family Practice"[MeSH] OR "family practice"[tiab] OR "Physicians, Family"[MeSH] OR "family physician*"[tiab] OR "family medicine"[tiab] | 343,256 |
| 2 | "digital health"[tiab] OR "digital health intervention"[tiab] OR "digital behavior change"[tiab] OR "digital behaviour change"[tiab] OR "digital health technology"[tiab] OR "Electronic Health Records"[MeSH] OR "electronic health record*"[tiab] OR "personal health record*"[tiab] OR "electronic medical record*"[tiab] OR "EMR"[tiab] OR "EHR"[tiab] OR "Health Records, Personal"[MeSH] OR "Patient Portals"[Mesh] OR "patient web portal*"[tiab] OR "patient web-portal*"[tiab] OR "patient portal*"[tiab] OR "web portal*"[tiab] OR "mobile technolog*"[tiab] OR "Telemedicine"[Mesh] OR "telemedicine"[tiab ] OR "telehealth*"[tiab] OR "mobile health"[tiab] OR "mHealth"[tiab] OR "eHealth"[tiab] OR "m-Health"[tiab] OR "mobile-health"[tiab] OR "telecommunication*"[tiab] OR ((app OR application*) n3 (smartphone* or smart-phone or mobile* or phone*)) OR "Decision Support Systems, Clinical"[Mesh] OR "clinical decision support"[tiab] OR "decision support system"[tiab] OR "Health Information Exchange"[MeSH] OR "health information exchange*"[tiab] OR "electronic health information"[tiab] OR "electronic health communication*"[tiab] OR "health information interoperability"[MeSH] OR "interoperability"[tw] OR "patient monitor*"[tiab] OR "wearables"[tiab] OR "activity monitor*"[tiab] OR "sensor*"[tiab] OR "Artificial Intelligence"[Mesh] OR "artificial intelligence"[tiab] OR "machine intelligence"[tiab] OR "computational intelligence"[tiab] OR "Machine Learning"[MeSH] OR "machine learning"[tiab] OR "machine-learning"[tiab] OR "natural language processing"[tiab] OR "neural network*"[tiab] OR "quantified self"[tiab] OR "connected health"[tiab] OR "big data"[tiab] OR "gamification"[tiab] OR "social media"[tiab] OR "health 2.0"[tiab] OR "internet of things"[tiab] OR "IoT"[tiab] OR "IOT"[tiab] OR (("social program*"[tiab] OR "care manage*"[tiab] OR "coordination care"[tiab:~2] OR "health benefit*"[tiab] OR insur*[tiab]) AND "digital"[tiab]) | 873,047 |
| 3 | #1 AND #2 | 19,238 |
| 4 | "Medical Informatics"[Mesh] OR "Data Science"[Mesh] OR "data analytics"[tiab] | 495,326 |
| 5 | #1 AND #4 | 13,705 |
| 6 | #3 OR #5 | 29,325 |
| 7 | "Preventive Medicine"[Mesh] OR "prevention and control "[subheading] OR "prevention"[tiab] OR "preventive"[tiab] OR "mass screening"[MeSH] OR "screening"[tiab] OR "preventive health services"[MeSH] OR "Patient Care Management"[MeSH] OR "care management"[tiab] OR "care management"[tiab] OR "comprehensive care"[tiab] OR "care planning"[tiab] or "disease management"[tiab] | 3,630,029 |
| 8 | #6 AND #7 | 23,275 |
| 9 | #6 AND #7 | English |
| 10 | #9 NOT (address[pt] OR "autobiography"[pt] OR "bibliography"[pt] OR "biography"[pt] OR "case control"[tw] OR "case report"[tw] OR "case reports"[tw] OR "case series"[tw] OR "comment"[pt] OR "comment on"[All Fields] OR congress[pt] OR "dictionary"[pt] OR "directory"[pt] OR "editorial"[pt] OR "festschrift"[pt] OR "historical article"[pt] OR "interview"[pt] OR lecture[pt] OR "legal case"[pt] OR "legislation"[pt] OR letter[pt] OR "news"[pt] OR "newspaper article"[pt] OR "patient education handout"[pt] OR "periodical index"[pt] OR ("Animals"[Mesh] NOT "Humans"[Mesh]) OR rats[tw] OR cow[tw] OR cows[tw] OR chicken[tw] OR chickens[tw] OR horse[tw] OR horses[tw] OR mice[tw] OR mouse[tw] OR bovine[tw] OR sheep OR ovine OR murine OR murinae) | 20,808 |
| 11 | "diagnosis"[MeSH] OR "diagnos*"[tiab] OR "diagnosis"[subheading] or "diagnostic"[tiab] | 11,501,547 |
| 12 | #10 NOT #11 | 12,427 |
| 13 | #10 NOT #11 AND from 2020-2023 | 3,158 |
| 14 | #13 AND ("Randomized Controlled Trial"[Publication Type] OR "Single-Blind Method"[MeSH] OR "Double-Blind Method"[MeSH] OR "Random Allocation"[MeSH] OR placebo[tiab] OR randomized[tiab] OR randomly[tiab] OR trial[tiab]) | 485 |
| 15 | #13 AND (("review"[Publication Type] AND "systematic"[tiab]) OR "systematic review"[All Fields] OR ("review literature as topic"[MeSH] AND "systematic"[tiab]) OR "meta-analysis"[Publication Type] OR "meta-analysis as topic"[MeSH Terms] OR "meta-analysis"[All Fields] OR "scoping review"[tiab:~2]) | 152 |
| 16 | #13 AND ("Cohort Studies"[Mesh] OR cohort OR (follow-up or followup) OR longitudinal OR "Research Design"[Mesh] OR "Evaluation Study" [Publication Type] OR "Comparative Study" [Publication Type] OR ((comparative or Intervention) AND study) OR pretest* OR posttest* OR prepost* OR "before and after" OR interrupted time* OR time serie* OR intervention* OR ((quasi-experiment* OR quasiexperiment* OR quasi or experimental) and (method or study or trial or design*)) OR (("real world" OR "real-world") AND (study or design))) | 2,369 |
| 17 | #14 OR #15 | 610 |
| 18 | #14 OR #15 OR #16 | 2,406 |
| 19 | #13 AND (access OR uptake OR understand* OR "patient physician"[tiab:~3] OR "patient education"[tiab:~3) | 1,277 |
| 20 | #18 AND #19 | 971 |
| 21 | #17 OR #20 | 1,341 |

Technology: Grey Literature Sources Searched

- AHRQ Digital Healthcare Research Program: <https://digital.ahrq.gov/>
- AHRQ Clinical Decision Support research program: <https://cds.ahrq.gov/>
- AHRQ: Health Assessments in Primary Care: A How-to Guide for Clinicians and Staff. 2014: <https://www.researchgate.net/publication/268332041_Health_Assessments_in_Primary_Care_A_How-to_Guide_for_Clinicians_and_Staff>
- AHRQ: Integrating Patient-Generated Health Data into Electronic Health Records in Ambulatory Care Settings: A Practical Guide: <https://digital.ahrq.gov/sites/default/files/docs/citation/pghd-practical-guide.pdf>
- AHRQ Question Builder: <https://www.ahrq.gov/questions/question-builder/online.html>
- Apple Health app: <https://www.apple.com/healthcare/health-records/>
- CDC: Goetzel RZ, Staley P, Ogden L, et al. A framework for patient-centered health risk assessments: providing health promotion and disease prevention services to Medicare beneficiaries. 2011: <https://www.cdc.gov/policy/paeo/hra/frameworkforhra.pdf>
- CommonHealth: <https://www.commonhealth.org/>
- Medicare Connected Apps Directory: <https://www.medicare.gov/manage-your-health/medicares-blue-button-blue-button-20/blue-button-apps>
- MyHealthfinder: <https://health.gov/myhealthfinder>
- Navigating Wellness: <https://navigatingwellness.org/>
- Office of the National Coordinator for Health Information Technology: HealthIT Playbook: <https://www.healthit.gov/playbook/>
- Prevention TaskForce: <https://www.uspreventiveservicestaskforce.org/apps/>

Technology: Technical Expert Panel Participants and Key Informants

The list of people who participated in the technical expert panel (TEP) or as key informants for the technology focus area is provided in Table A-4.

Table A-4. Technology Technical Expert Panel Members and Key Informants

| Name | Organization | Role | Type of Organization |
| --- | --- | --- | --- |
| Benjamin Broder, MD, PhD | Kaiser Permanente, Southern California Permanente Medical Group | Senior Director of Research; Regional Assistant Medical Director | Healthcare system |
| Melony Burnett | Kaiser Permanente’s Patient Partner Volunteer/Patient Advisory Council | Member | Patient/consumer representative |
| Stacie Carney, MD | OCHIN—nonprofit innovation center providing health IT support services | Chief Medical Information Officer | Health information technology |
| Shannon Dowler, MD, FAAFP, CRE | State of North Carolina Department of Health and Human Services; Medicaid | Assistant Secretary for Health Access; Chief Medical Officer NC Medicaid | Payers |
| Robert Jarrin, JD | The Omega Concern, LLC | Managing Member | Research/academia |
| Ryan Jelinek, DO | Hennepin Healthcare | Medical Director for Telehealth and Patient Access | Healthcare system |
| Tom Keane, MD, MBA | Office of the National Coordinator for Health Information Technology (ONC) | Former Senior Advisor at ONC | Federal agency |
| Alex Krist, MD, MPH | Virginia Commonwealth University | Professor and Co-Director, Ambulator Care Outcomes | USPSTF (current/former member) |
| Edna Leed | Kaiser Permanente's Person and Family Centered Care Program | Member | Patient/consumer representative |
| Ed Lomotan, MD | Agency for Healthcare Research and Quality | Senior Advisor for Clinical Informatics | Federal agency |
| John Ruiz, PhD | University of Arizona, Department of Psychology | Professor and Director of Diversity, Equity, and Inclusion | USPSTF (current/former member) |
| Matthew Sakumoto, MD | Sutter Health | Virtualist Primary Care | AHRQ Primary Care Learning Community |
| Julia Skapik MD, MPH | National Association of Community Health Centers | Chief Medical Information Officer | Healthcare system |
| Glen Taksler, PhD | Cleveland Clinic Community Care | Associate Professor of Medicine | Research/academia |
| Anonymous | - | - | 1 from a Healthcare system  1 from field of Health information technology |

Technology: Technical Expert Panel and Key Informant Interview Questions

- What challenges do patients face when using these kinds of technologies?
- What makes them easier for patients to use?
- What challenges do providers and health systems face when using these kinds of technologies?
- What makes them easier for providers and health systems to use?
- What areas or specific technologies have the most potential?
- What new technologies are on the horizon in this space?
- From your perspective, where is more work needed?
  - In technology development?
  - In integration of care?
  - In the use of technology in CPS?

Innovative Delivery Models: Environmental Scan Data Sources and Searches

Searches of electronic databases were limited to PubMed, CINAHL, and the Cochrane Library (**Table A-5**). The searches covered the period from January 1, 2012, to February 7, 2023. Grey literature sources were also searched. All citations were managed and deduplicated using EndNote X9 (Clarivate Analytics). DistillerSR was used to manage the abstract and article screening and review process.

**Table A-5**. Search Strategy for the Innovative Delivery Models Topic Area

| Search Number | PubMed Query | Filters | Results |
| --- | --- | --- | --- |
| 1 | ("Primary Health Care"[Majr] OR "Physicians, Primary Care"[Majr] OR primary care[title] OR "primary health care"”[title] OR PHC[title] OR "General Practice"[Majr] OR "Family Practice"[Majr] OR "general practice"[title] OR "family practice"[title] OR "Preventive Health Services"[Majr] OR "Preventive Medicine"[Majr] OR "General Practice"[Majr] OR "general practice"[title] OR "family practice"[title] OR "Preventive Health Services"[Majr] OR "Preventive Medicine"[Majr] OR "Community Health Centers"[Majr] OR "Federally Qualified Health Center"[title] OR "Federally Qualified Health Centers"[title] OR FQHC*[title]) |  | [569,947](https://pubmed.ncbi.nlm.nih.gov/?term=%28%22Primary+Health+Care%22%5BMajr%5D+OR+%22Physicians%2C+Primary+Care%22%5BMajr%5D+OR+%22primary+care%22%5Btitle%5D+OR+%22primary+health+care%22%5Btitle%5D+OR+PHC%5Btitle%5D+OR+%22General+Practice%22%5BMajr%5D+OR+%22Family+Practice%22%5BMajr%5D+OR+%22general+practice%22%5Btitle%5D+OR+%22family+practice%22%5Btitle%5D+OR+%22Preventive+Health+Services%22%5BMajr%5D+OR+%22Preventive+Medicine%22%5BMajr%5D+OR+%22General+Practice%22%5BMajr%5D+OR+%22general+practice%22%5Btitle%5D+OR+%22family+practice%22%5Btitle%5D+OR+%22Preventive+Health+Services%22%5BMajr%5D+OR+%22Preventive+Medicine%22%5BMajr%5D+OR+%22Community+Health+Centers%22%5BMajr%5D+OR+%22Federally+Qualified+Health+Center%22%5Btitle%5D+OR+%22Federally+Qualified+Health+Centers%22%5Btitle%5D+OR+FQHC%2A%5Btitle%5D%29&sort=relevance&size=50&ac=no) |
| 2 | "Public Health"[Majr] OR "Public Health Administration"[Majr] OR "Public Health Nursing"[Majr] OR "Public Health Surveillance"[Majr] OR "public health"[title] OR "Population Health"[Majr] OR "population health"[title] |  | [2,105,736](https://pubmed.ncbi.nlm.nih.gov/?term=%22Public+Health%22%5BMajr%5D+OR+%22Public+Health+Administration%22%5BMajr%5D+OR+%22Public+Health+Nursing%22%5BMajr%5D+OR+%22Public+Health+Surveillance%22%5BMajr%5D+OR+%22public+health%22%5Btitle%5D+OR+%22Population+Health%22%5BMajr%5D+OR+%22population+health%22%5Btitle%5D&sort=relevance&size=50&ac=no) |
| 3 | #1 AND #2 |  | [219,757](https://pubmed.ncbi.nlm.nih.gov/?term=%231+AND+%232&sort=relevance&size=50&ac=no) |
| 4 | "Community based organization*"[tiab] OR church*[title] OR "Faith-Based Organizations"[Majr] OR "community engagement"[title] OR "community-based organisation"[title] OR "community based organisations"[title] OR "Community-Based Participatory Research"[Majr] OR "community-based participatory research"[title] OR "Community-based program"[title] OR "community-based programs"[title] OR CBPR[tiab] OR "Clinic-community linkages"[tiab] |  | [10,750](https://pubmed.ncbi.nlm.nih.gov/?term=%22Community+based+organization%2A%22%5Btiab%5D+OR+church%2A%5Btitle%5D+OR+%22Faith-Based+Organizations%22%5BMajr%5D+OR+%22community+engagement%22%5Btitle%5D+OR+%22community-based+organisation%22%5Btitle%5D+OR+%22community+based+organisations%22%5Btitle%5D+OR+%22Community-Based+Participatory+Research%22%5BMajr%5D+OR+%22community-based+participatory+research%22%5Btitle%5D+OR+%22Community-based+program%22%5Btitle%5D+OR+%22community-based+programs%22%5Btitle%5D+OR+CBPR%5Btiab%5D+OR+%22Clinic-community+linkages%22%5Btiab%5D&sort=relevance&size=50&ac=no) |
| 5 | #1 AND #4 |  | [1,793](https://pubmed.ncbi.nlm.nih.gov/?term=%231+AND+%234&sort=relevance&size=50&ac=no) |
| 6 | #3 OR #5 |  | [221,073](https://pubmed.ncbi.nlm.nih.gov/?term=%233+OR+%235&sort=relevance&size=50&ac=no) |
| 7 | "Models, Organizational"[Majr] OR "Organizational Innovation"[Majr] OR Collaboration[title] OR "Health Facility Merger"[Majr] OR "Systems Integration"[Majr] OR "Cooperative Behavior"[Majr] OR Integration[title] OR integrated[title] OR "organizational model*"[title] OR "collaborative"[title] OR Partnership[title] OR partner[title] OR partners[title] OR linkage*[title/abstract] OR cooperation[title] OR integration[title] |  | [344,809](https://pubmed.ncbi.nlm.nih.gov/?term=%22Models%2C+Organizational%22%5BMajr%5D+OR+%22Organizational+Innovation%22%5BMajr%5D+OR+Collaboration%5Btitle%5D+OR+%22Health+Facility+Merger%22%5BMajr%5D+OR+%22Systems+Integration%22%5BMajr%5D+OR+%22Cooperative+Behavior%22%5BMajr%5D+OR+Integration%5Btitle%5D+OR+integrated%5Btitle%5D+OR+%22organizational+model%2A%22%5Btitle%5D+OR+%22collaborative%22%5Btitle%5D+OR+Partnership%5Btitle%5D+OR+partner%5Btitle%5D+OR+partners%5Btitle%5D+OR+linkage%2A%5Btitle%2Fabstract%5D+OR+cooperation%5Btitle%5D+OR+integration%5Btitle%5D&sort=relevance&size=50&ac=no) |
| 8 | #6 AND #7 |  | [3,924](https://pubmed.ncbi.nlm.nih.gov/?term=%236+AND+%237&sort=relevance&size=50&ac=no) |
| 9 | "Delivery of Health Care"[Majr] OR "Delivery of Health Care, Integrated"[Majr] OR "Patient Care Team"[Majr] OR "Continuity of Patient Care"[Majr] OR "Patient-Centered Care"[Mesh] OR "Patient Centered"[title] OR "Person centered"[title] OR "Care coordination"[title:~2] OR "coordinated care"[title:~2] |  | [921,237](https://pubmed.ncbi.nlm.nih.gov/?term=%22Delivery+of+Health+Care%22%5BMajr%5D+OR+%22Delivery+of+Health+Care%2C+Integrated%22%5BMajr%5D+OR+%22Patient+Care+Team%22%5BMajr%5D+OR+%22Continuity+of+Patient+Care%22%5BMajr%5D+OR+%22Patient-Centered+Care%22%5BMesh%5D+OR+%22Patient+Centered%22%5Btitle%5D+OR+%22Person+centered%22%5Btitle%5D+OR+%22Care+coordination%22%5Btitle%3A~2%5D+OR+%22coordinated+care%22%5Btitle%3A~2%5D&sort=relevance&size=50&ac=no) |
| 10 | #8 AND #9 |  | [1,137](https://pubmed.ncbi.nlm.nih.gov/?term=%238+AND+%239&sort=relevance&size=50&ac=no) |
| 11 | "Preventive Health Services"[Mesh] OR "clinical preventive services"[tiab] OR "clinical preventive service"[tiab] OR Preventive[title] OR Prevention[title] OR Mass Screening[Mesh] OR screening[title] OR screen*[title] OR screens[title] OR screened[title] OR Counseling[Mesh] OR counsel*[title] OR counseling[title] OR Immunization[Mesh] OR Vaccination[Mesh] OR "Immunization Programs"[Mesh] OR immunization[title] OR vaccine*[title] OR vaccination*[title] OR "Primary Prevention"[Majr] OR "Chronic Disease/prevention and control"[Mesh] |  | [1,211,923](https://pubmed.ncbi.nlm.nih.gov/?term=%22Preventive+Health+Services%22%5BMesh%5D+OR+%22clinical+preventive+services%22%5Btiab%5D+OR+%22clinical+preventive+service%22%5Btiab%5D+OR+Preventive%5Btitle%5D+OR+Prevention%5Btitle%5D+OR+Mass+Screening%5BMesh%5D+OR+screening%5Btitle%5D+OR+screen%2A%5Btitle%5D+OR+screens%5Btitle%5D+OR+screened%5Btitle%5D+OR+Counseling%5BMesh%5D+OR+counsel%2A%5Btitle%5D+OR+counseling%5Btitle%5D+OR+Immunization%5BMesh%5D+OR+Vaccination%5BMesh%5D+OR+%22Immunization+Programs%22%5BMesh%5D+OR+immunization%5Btitle%5D+OR+vaccine%2A%5Btitle%5D+OR+vaccination%2A%5Btitle%5D+OR+%22Primary+Prevention%22%5BMajr%5D+OR+%22Chronic+Disease%2Fprevention+and+control%22%5BMesh%5D&sort=relevance&size=50&ac=no) |
| 12 | #8 AND #11 |  | [2,933](https://pubmed.ncbi.nlm.nih.gov/?term=%238+AND+%2311&sort=relevance&size=50&ac=no) |
| 13 | #10 OR #12 |  | [3,412](https://pubmed.ncbi.nlm.nih.gov/?term=%2310+OR+%2312&sort=relevance&size=50&ac=no) |
| 14 | Intervention*[tw] OR program[tiab] OR programs[tiab] OR "Evaluation Study"[PT] OR "Evaluation Studies as Topic"[Mesh] OR "Program Evaluation"[Mesh] OR Evaluation[ti] OR "Health Plan Implementation"[Mesh] OR "Health Impact Assessment"[Mesh] OR "Patient Outcome Assessment"[Mesh] |  | [3,630,275](https://pubmed.ncbi.nlm.nih.gov/?term=Intervention%2A%5Btw%5D+OR+program%5Btiab%5D+OR+programs%5Btiab%5D+OR+%22Evaluation+Study%22%5BPT%5D+OR+%22Evaluation+Studies+as+Topic%22%5BMesh%5D+OR+%22Program+Evaluation%22%5BMesh%5D+OR+Evaluation%5Bti%5D+OR+%22Health+Plan+Implementation%22%5BMesh%5D+OR+%22Health+Impact+Assessment%22%5BMesh%5D+OR+%22Patient+Outcome+Assessment%22%5BMesh%5D&sort=relevance&size=50&ac=no) |
| 15 | #13 AND #14 |  | [1,739](https://pubmed.ncbi.nlm.nih.gov/?term=%2313+AND+%2314&sort=relevance&size=50&ac=no) |
| 16 | #13 AND #14 | English | [1,662](https://pubmed.ncbi.nlm.nih.gov/?term=%2313+AND+%2314&filter=lang.english&ac=no&size=50&sort=relevance) |
| 17 | #13 AND #14 | English, from 2012 – 2023 | [1,092](https://pubmed.ncbi.nlm.nih.gov/?term=%2313+AND+%2314&filter=lang.english&filter=years.2012-2023&ac=no&size=50&sort=relevance) |
| 18 | ("Animals"[Mesh] NOT "Humans"[Mesh]) OR rats[tw] OR cow[tw] OR cows[tw] OR chicken[tw] OR chickens[tw] OR horse[tw] OR horses[tw] OR mice[tw] OR mouse[tw] OR bovine[tw] OR sheep[tw] OR ovine[tw] OR murine[tw] OR murinae[tw] |  | [6,379,783](https://pubmed.ncbi.nlm.nih.gov/?term=%28%22Animals%22%5BMesh%5D+NOT+%22Humans%22%5BMesh%5D%29+OR+rats%5Btw%5D+OR+cow%5Btw%5D+OR+cows%5Btw%5D+OR+chicken%5Btw%5D+OR+chickens%5Btw%5D+OR+horse%5Btw%5D+OR+horses%5Btw%5D+OR+mice%5Btw%5D+OR+mouse%5Btw%5D+OR+bovine%5Btw%5D+OR+sheep%5Btw%5D+OR+ovine%5Btw%5D+OR+murine%5Btw%5D+OR+murinae%5Btw%5D&sort=relevance&size=50&ac=no) |
| 19 | #17 NOT #18 |  | [1,085](https://pubmed.ncbi.nlm.nih.gov/?term=%2317+NOT+%2318&sort=relevance&size=50&ac=no) |
| 20 | #17 NOT #18 | Systematic Review | [31](https://pubmed.ncbi.nlm.nih.gov/?term=%2317+NOT+%2318&filter=pubt.systematicreview&ac=no&size=50&sort=relevance) |
| 21 | #17 NOT #18 | Meta-Analysis, Systematic Review | [35](https://pubmed.ncbi.nlm.nih.gov/?term=%2317+NOT+%2318&filter=pubt.meta-analysis&filter=pubt.systematicreview&ac=no&size=50&sort=relevance) |
| 22 | #19 AND ("scoping review"[tiab] OR "integrative review"[tiab] OR "rapid review"[tiab] OR "living review"[tiab] OR "environmental scan"[tiab]) |  | [14](https://pubmed.ncbi.nlm.nih.gov/?term=%2319+AND+%28%22scoping+review%22%5Btiab%5D+OR+%22integrative+review%22%5Btiab%5D+OR+%22rapid+review%22%5Btiab%5D+OR+%22living+review%22%5Btiab%5D+OR+%22environmental+scan%22%5Btiab%5D%29&sort=relevance&size=50&ac=no) |
| 23 | #22 NOT #21 |  | [11](https://pubmed.ncbi.nlm.nih.gov/?term=%2322+NOT+%2321&sort=relevance&size=50&ac=no) |
| 24 | #19 NOT (#21 OR #23) |  | [1,039](https://pubmed.ncbi.nlm.nih.gov/?term=%2319+NOT+%28%2321+OR+%2323%29&sort=relevance&size=50&ac=no) |
| 25 | toolkit[tw] OR toolkits[tw] OR "tool kit"[tw] OR "tool kits"[tw] |  | [11,429](https://pubmed.ncbi.nlm.nih.gov/?term=toolkit%5Btw%5D+OR+toolkits%5Btw%5D+OR+%22tool+kit%22%5Btw%5D+OR+%22tool+kits%22%5Btw%5D&sort=relevance&size=50&ac=no) |
| 26 | #19 AND #25 |  | [4](https://pubmed.ncbi.nlm.nih.gov/?term=%2319+AND+%2325&sort=relevance&size=50&ac=no) |

| Search Number | Cochrane Library Query | Filters | Results |
| --- | --- | --- | --- |
| 1 | [mh "Patient-Centered Care"] OR ((patient-centered:ti,ab OR patient-focused:ti,ab OR person-centered:ti,ab) AND care:ti,ab) OR [mh "Precision Medicine"] OR ((individual*:ti,ab OR Individualize*:ti,ab OR holistic:ti,ab OR "whole person":ti,ab OR personalized:ti,ab) AND care:ti,ab) |  | 38,115 |
| 2 | "patient needs":ti,ab OR "patient values":ti,ab OR [mh "Physician-Patient Relations"] OR ("Doctor-patient" NEXT relation*):ti,ab OR [mh "Professional-Patient Relations"] OR [mh "Patient Preference"] OR ("patient" NEXT preference*):ti,ab OR ("social" NEXT competenc*):ti,ab OR [mh "Decision Making, Shared"] OR "shared decision making":ti,ab OR [mh "Patient Self-Determination Act"] OR "patient decision making":ti,ab OR "patient engagement":ti,ab OR "patient involvement":ti,ab OR "patient empowerment":ti,ab OR "patient partnership":ti,ab OR "patient activation":ti,ab OR “patient-activated”:ti,ab OR [mh "Patient Acceptance of Health Care"] OR "consumer participation":ti OR "consumer engagement":ti,ab OR "consumer involvement":ti,ab OR "consumer empowerment":ti,ab OR "consumer partnership":ti,ab OR "consumer activation":ti,ab OR "patient context":ti,ab OR "integrated care":ti,ab OR "coordinated care":ti,ab OR "Care coordination":ti,ab OR "continuity of care":ti,ab OR "healthcare teams":ti,ab OR "team-based care":ti,ab OR teamwork:ti,ab |  | 34,005 |
| 3 | #1 OR #2 |  | 68,096 |
| 4 | [mh "Preventive Health Services"] OR "clinical preventive services":ti,ab OR "clinical preventive service":ti,ab OR Preventive:ti OR Prevention:ti OR [mh “Mass Screening”] OR screening:ti OR screen*:ti OR screens:ti OR screened:ti OR [mh Counseling] OR counsel*:ti OR counseling:ti OR [mh Immunization] OR [mh Vaccination] OR [mh "Immunization Programs"] OR immunization:ti OR vaccine*:ti OR vaccination*:ti OR [mh "Primary Prevention"] |  | 119,457 |
| 5 | #3 AND #4 |  | 12,083 |
| 6 | [mh "Primary Health Care"] OR [mh "Physicians, Primary Care"] OR "primary care":ti OR "primary health care":ti OR PHC:ti OR [mh "General Practice"] OR [mh "Family Practice"] OR "general practice":ti OR "family practice":ti OR [mh "Preventive Health Services"] OR [mh "Preventive Medicine"] OR [mh "Community Health Centers"] OR "Federally Qualified Health Center":ti OR "Federally Qualified Health Centers":ti OR FQHC*:ti OR "healthcare center":ti,ab OR "healthcare clinic":ti,ab OR "healthcare clinics":ti,ab OR ("healthcare" NEXT system*):ti,ab OR ("healthcare" NEXT organization*):ti,ab OR ("community health" NEXT worker*):ti,ab OR navigator*:ti,ab OR peers:ti,ab OR peer:ti,ab |  | 72,509 |
| 7 | [mh "Organizational Innovation"] OR [mh "Models, Organizational"] OR ("care" NEXT model*):ti,ab OR service:ti,ab OR ([mh "Delivery of Health Care"] AND model*:ti,ab,kw) OR program:ti,ab OR programmatic:ti,ab OR redesign:ti,ab OR transformation:ti,ab OR innovation:ti,ab OR innovative:ti,ab OR "new model":ti,ab OR reform:ti OR quality:ti OR "healthcare improvement":ti,ab OR "system improvement":ti,ab OR strategy:ti OR strategies:ti OR "improve care":ti OR "care improvement":ti OR "care delivery":ti OR [mh "Health Systems Agencies"] OR [mh "Social Determinants of Health"] OR [mh "Patient Care Bundles"] OR [mh "Patient Reported Outcome Measures"] OR ("Quality" NEXT Measure*):ti,ab OR [mh "Healthy People Programs"] |  | 199,028 |
| 8 | #6 AND #7 |  | 27,135 |
| 9 | #5 AND #8 |  | 4,123 |
| 10 | #9 NOT ([mh Animals] NOT [mh Humans]) |  | 4,123 |
| 11 | #10 NOT (([mh Adolescent] OR [mh Child] OR [mh Infant]) NOT [mh Adult]) |  | 3,764 |
| 12 | #11 NOT (framework*:ti,ab OR concept:ti,ab OR theoretical:ti,ab) |  | 3,564 |
| 13 | [mh "afghanistan"] OR [mh africa] OR [mh "africa, northern"] OR [mh "africa, central"] OR [mh "africa, eastern"] OR [mh "africa south of the sahara"] OR [mh "africa, southern"] OR [mh "africa, western"] OR [mh albania] OR [mh algeria] OR [mh andorra] OR [mh angola] OR [mh "antigua and barbuda"] OR [mh argentina] OR [mh armenia] OR [mh azerbaijan] OR [mh bahamas] OR [mh bahrain] OR [mh bangladesh] OR [mh barbados] OR [mh belize] OR [mh benin] OR [mh bhutan] OR [mh bolivia] OR [mh borneo] OR [mh "bosnia and herzegovina"] OR [mh botswana] OR [mh brazil] OR [mh brunei] OR [mh bulgaria] OR [mh "burkina faso"] OR [mh burundi] OR [mh "cabo verde"] OR [mh cambodia] OR [mh cameroon] OR [mh "central african republic"] OR [mh chad] OR [mh china] OR [mh comoros] OR [mh congo] OR [mh croatia] OR [mh cuba] OR [mh "democratic republic of the congo"] OR [mh cyprus] OR [mh djibouti] OR [mh dominica] OR [mh "dominican republic"] OR [mh ecuador] OR [mh egypt] OR [mh "el salvador"] OR [mh "equatorial guinea"] OR [mh eritrea] OR [mh eswatini] OR [mh ethiopia] OR [mh fiji] OR [mh gabon] OR [mh gambia] OR [mh "georgia (republic)"] OR [mh ghana] OR [mh grenada] OR [mh guatemala] OR [mh guinea] OR (guinea AND bissau) OR [mh guyana] OR [mh haiti] OR [mh honduras] OR [mh "independent state of samoa"] OR [mh india] OR [mh "indian ocean islands"] OR [mh indochina] OR [mh indonesia] OR [mh iran] OR [mh iraq] OR [mh jamaica] OR [mh jordan] OR [mh kazakhstan] OR [mh kenya] OR [mh kosovo] OR [mh kuwait] OR [mh kyrgyzstan] OR [mh laos] OR [mh lebanon] OR [mh liechtenstein] OR [mh lesotho] OR [mh liberia] OR [mh libya] OR [mh madagascar] OR [mh malaysia] OR [mh malawi] OR [mh mali] OR [mh malta] OR [mh mauritania] OR [mh mauritius] OR [mh "mekong valley"] OR [mh melanesia] OR [mh micronesia] OR [mh monaco] OR [mh mongolia] OR [mh montenegro] OR [mh morocco] OR [mh mozambique] OR [mh myanmar] OR [mh namibia] OR [mh nepal] OR [mh nicaragua] OR [mh niger] OR [mh nigeria] OR [mh oman] OR [mh pakistan] OR [mh palau] OR [mh panama] OR [mh "papua new guinea"] OR [mh paraguay] OR [mh peru] OR [mh philippines] OR [mh qatar] OR [mh "republic of belarus"] OR [mh "republic of north macedonia"] OR [mh romania] OR [mh russia] OR [mh rwanda] OR [mh "saint kitts and nevis"] OR [mh "saint lucia"] OR [mh "saint vincent and the grenadines"] OR [mh "sao tome and principe"] OR [mh "saudi arabia"] OR [mh serbia] OR [mh "sierra leone"] OR [mh senegal] OR [mh seychelles] OR [mh singapore] OR [mh somalia] OR [mh "south sudan"] OR [mh "sri lanka"] OR [mh sudan] OR [mh suriname] OR [mh syria] OR [mh taiwan] OR [mh tajikistan] OR [mh tanzania] OR [mh thailand] OR (timor AND leste) OR [mh togo] OR [mh tonga] OR [mh "trinidad and tobago"] OR [mh tunisia] OR [mh turkmenistan] OR [mh uganda] OR [mh ukraine] OR [mh "united arab emirates"] OR [mh uruguay] OR [mh uzbekistan] OR [mh vanuatu] OR [mh venezuela] OR [mh vietnam] OR [mh "west indies"] OR [mh yemen] OR [mh zambia] OR [mh zimbabwe] |  | 34,570 |
| 14 | (“Organisation for Economic” NEAR Development) OR [mh "European Union"] OR [mh "Developed Countries"] OR [mh australasia] OR [mh australia] OR [mh austria] OR [mh "baltic states"] OR [mh belgium] OR [mh canada] OR [mh chile] OR [mh colombia] OR [mh "costa rica"] OR [mh "czech republic"] OR [mh denmark] OR [mh estonia] OR [mh europe] OR [mh finland] OR [mh france] OR [mh germany] OR [mh greece] OR [mh hungary] OR [mh iceland] OR [mh ireland] OR [mh israel] OR [mh italy] OR [mh japan] OR [mh korea] OR [mh latvia] OR [mh lithuania] OR [mh luxembourg] OR [mh mexico] OR [mh netherlands] OR [mh "new zealand"] OR [mh "north america"] OR [mh norway] OR [mh poland] OR [mh portugal] OR [mh "republic of korea"] OR [mh "scandinavian and nordic countries"] OR [mh slovakia] OR [mh slovenia] OR [mh spain] OR [mh sweden] OR [mh switzerland] OR [mh turkey] OR [mh "united kingdom"] OR [mh "united states"] |  | 80,484 |
| 15 | #13 NOT #14 |  | 32,213 |
| 16 | #12 NOT #15 |  | 3,221 |
| 17 | Limited to Cochrane Reviews published 2012-2023 |  | 61 |

| **Search Number** | **CINAHL Search Query** | **Limiters/Expanders** | **Results** |
| --- | --- | --- | --- |
| 1 | (MH "Patient-Centered Care+") OR (((TI patient-centered OR AB patient-centered) OR (TI patient-focused OR AB patient-focused) OR (TI person-centered OR AB person-centered)) AND (TI care OR AB care)) OR (MM "Precision Medicine+") OR (((TI individual* OR AB individual*) OR (TI Individualize* OR AB Individualize*) OR (TI holistic OR AB holistic) OR (TI "whole person" OR AB "whole person") OR (TI personalized OR AB personalized)) AND (TI care OR AB care)) | Expanders - Apply equivalent subjects Search modes - Find all my search terms | 130,614 |
| 2 | (TI " "patient needs" OR AB ""patient needs") OR (TI "patient values" OR AB "patient values") OR (MM "Physician-Patient Relations+") OR (TI "Doctor-patient relation*" OR AB "Doctor-patient relation*") OR (MM "Professional-Patient Relations+") OR (MM "Patient Preference+") OR (TI "patient preference*" OR AB "patient preference*") OR (TI "social competenc*" OR AB "social competenc*") OR (MM "Decision Making, Shared+") OR (TI "shared decision making" OR AB "shared decision making") OR (MM "Patient Self-Determination Act+") OR (TI "patient decision making" OR AB "patient decision making") OR (TI "patient engagement" OR AB "patient engagement") OR (TI "patient involvement" OR AB "patient involvement") OR (TI "patient empowerment" OR AB "patient empowerment") OR (TI "patient partnership" OR AB "patient partnership") OR (TI "patient activation" OR AB "patient activation") OR (TI patient-activated OR AB patient-activated) OR (MM "Patient Acceptance of Health Care+") OR (TI "consumer participation") OR (TI "consumer engagement" OR AB "consumer engagement") OR (TI "consumer involvement" OR AB "consumer involvement") OR (TI "consumer empowerment" OR AB "consumer empowerment") OR (TI "consumer partnership" OR AB "consumer partnership") OR (TI "consumer activation" OR AB "consumer activation") OR (TI "patient context" OR AB "patient context") OR (TI "integrated care" OR AB "integrated care") OR (TI "coordinated care" OR AB "coordinated care") OR (TI "Care coordination" OR AB "Care coordination") OR (TI "continuity of care" OR AB "continuity of care") OR (TI "healthcare teams" OR AB "healthcare teams") OR (TI "team-based care" OR AB "team-based care") OR (TI teamwork OR AB teamwork) | Expanders - Apply equivalent subjects Search modes - Find all my search terms | 95,133 |
| 3 | S1 OR S2 | Expanders - Apply equivalent subjects Search modes - Find all my search terms | 215,213 |
| 4 | (MM "Preventive Health Services+") OR (TI "clinical preventive services" OR AB "clinical preventive services") OR (TI "clinical preventive service" OR AB "clinical preventive service") OR (TI Preventive) OR (TI Prevention) OR (MM “Mass Screening+”) OR (TI screening) OR (TI screen*) OR (TI screens) OR (TI screened) OR (MM Counseling+) OR (TI counsel*) OR (TI counseling) OR (MM Immunization+) OR (MM Vaccination+) OR (MM "Immunization Programs+") OR (TI immunization) OR (TI vaccine*) OR (TI vaccination*) OR (MM "Primary Prevention+") | Expanders - Apply equivalent subjects Search modes - Find all my search terms | 240,084 |
| 5 | S3 AND S4 | Expanders - Apply equivalent subjects Search modes - Find all my search terms | 8,932 |
| 6 | (MM "Primary Health Care+") OR (MM "Physicians, Primary Care+") OR (TI "primary care") OR (TI "primary health care") OR (TI PHC) OR (MM "General Practice+") OR (MM "Family Practice+") OR (TI "general practice") OR (TI "family practice") OR (MM "Preventive Health Services+") OR (MM "Preventive Medicine+") OR (MH "Community Health Centers+") OR (TI "Federally Qualified Health Center") OR (TI "Federally Qualified Health Centers") OR (TI FQHC*) OR (TI "healthcare center" OR AB "healthcare center") OR (TI "healthcare clinic" OR AB "healthcare clinic") OR (TI "healthcare clinics" OR AB "healthcare clinics") OR (TI "healthcare system*" OR AB "healthcare system*") OR (TI "healthcare organization*" OR AB "healthcare organization*") OR (TI "community health worker*" OR AB "community health worker*") OR (TI navigator* OR AB navigator*) OR (TI peers OR AB peers) OR (TI peer OR AB peer) | Expanders - Apply equivalent subjects Search modes - Find all my search terms | 182,117 |
| 7 | (MM "Organizational Innovation+") OR (MM "Models, Organizational+") OR (TI "care model*" OR AB "care model*") OR (TI service OR AB service) OR ((MM "Delivery of Health Care+") AND model*) OR (TI program* OR AB program*) OR (TI programmatic OR AB programmatic) OR (TI redesign OR AB redesign) OR (TI transformation OR AB transformation) OR (TI innovation OR AB innovation) OR (TI innovative OR AB innovative) OR (TI "new model" OR AB "new model") OR (TI reform) OR (TI quality) OR (TI "healthcare improvement" OR AB "healthcare improvement") OR (TI "system improvement" OR AB "system improvement") OR (TI strategy) OR (TI strategies) OR (TI "improve care") OR (TI "care improvement") OR (TI "care delivery") OR (MM "Health Systems Agencies+") OR (MH "Social Determinants of Health+") OR (MH "Patient Care Bundles+") OR (MM "Patient Reported Outcome Measures+") OR (TI "Quality Measure*" OR AB "Quality Measure*") OR (MM "Healthy People Programs+") | Expanders - Apply equivalent subjects Search modes - Find all my search terms | 992,940 |
| 8 | S6 AND S7 | Expanders - Apply equivalent subjects Search modes - Find all my search terms | 61,193 |
| 9 | S5 AND S8 | Expanders - Apply equivalent subjects Search modes - Find all my search terms | 669 |
| 10 | S9 | Limiters - English Language; Human Expanders - Apply equivalent subjects Search modes - Find all my search terms | 468 |
| 11 | S10 | Limiters - Published Date: 20120101-20231231 Expanders - Apply equivalent subjects Search modes - Find all my search terms | 358 |
| 12 | (PT address) OR (PT autobiography) OR (PT bibliography) OR (PT biography) OR (PT congress) OR (PT dictionary) OR (PT directory) OR (PT festschrift) OR (PT "historical article") OR (PT lecture) OR (PT "legal case") OR (PT legislation) OR (PT "periodical index") OR rats OR cow OR cows OR chicken OR chickens OR horse OR horses OR mice OR mouse OR bovine OR sheep OR ovine OR murine OR murinae OR Self-management OR "disease management" OR protocol OR (TI hospital*) OR (TI discharge*) OR (TI transition*) | Limiters - Published Date: 20120101-20231231 Expanders - Apply equivalent subjects Search modes - Find all my search terms | 411,067 |
| 13 | S11 NOT S12 | Limiters - Published Date: 20120101-20231231 Expanders - Apply equivalent subjects Search modes - Find all my search terms | 310 |
| 14 | S13 NOT (((MH Adolescent+) OR (MH Child+) OR (MH Infant+)) NOT (MH Adult+)) | Limiters - Published Date: 20120101-20231231 Expanders - Apply equivalent subjects Search modes - Find all my search terms | 294 |
| 15 | S14 | Limiters - Age Groups: All Adult Expanders - Apply equivalent subjects Search modes - Find all my search terms | 173 |
| 16 | S15 NOT ((TI framework* OR AB framework*) OR (TI concept OR AB concept) OR (TI theoretical OR AB theoretical)) | Limiters - Age Groups: All Adult Expanders - Apply equivalent subjects Search modes - Find all my search terms | 159 |
| 17 | (MH afghanistan) OR (MH africa) OR (MH "africa, northern") OR (MH "africa, central") OR (MH "africa, eastern") OR (MH "africa south of the sahara") OR (MH "africa, southern") OR (MH "africa, western") OR (MH albania) OR (MH algeria) OR (MH andorra) OR (MH angola) OR (MH "antigua and barbuda") OR (MH argentina) OR (MH armenia) OR (MH azerbaijan) OR (MH bahamas) OR (MH bahrain) OR (MH bangladesh) OR (MH barbados) OR (MH belize) OR (MH benin) OR (MH bhutan) OR (MH bolivia) OR (MH borneo) OR (MH "bosnia and herzegovina") OR (MH botswana) OR (MH brazil) OR (MH brunei) OR (MH bulgaria) OR (MH "burkina faso") OR (MH burundi) OR (MH "cabo verde") OR (MH cambodia) OR (MH cameroon) OR (MH "central african republic") OR (MH chad) OR (MH china+) OR (MH comoros) OR (MH congo) OR (MH croatia) OR (MH cuba) OR (MH "democratic republic of the congo") OR (MH cyprus) OR (MH djibouti) OR (MH dominica) OR (MH "dominican republic") OR (MH ecuador) OR (MH egypt) OR (MH "el salvador") OR (MH "equatorial guinea") OR (MH eritrea) OR (MH eswatini) OR (MH ethiopia) OR (MH fiji) OR (MH gabon) OR (MH gambia) OR (MH "georgia (republic)") OR (MH ghana) OR (MH grenada) OR (MH guatemala) OR (MH guinea) OR (MH guinea-bissau) OR (MH guyana) OR (MH haiti) OR (MH honduras) OR (MH "independent state of samoa") OR (MH india+) OR (MH "indian ocean islands") OR (MH indochina) OR (MH indonesia) OR (MH iran) OR (MH iraq) OR (MH jamaica) OR (MH jordan) OR (MH kazakhstan) OR (MH kenya) OR (MH kosovo) OR (MH kuwait) OR (MH kyrgyzstan) OR (MH laos) OR (MH lebanon) OR (MH liechtenstein) OR (MH lesotho) OR (MH liberia) OR (MH libya) OR (MH madagascar) OR (MH malaysia) OR (MH malawi) OR (MH mali) OR (MH malta) OR (MH mauritania) OR (MH mauritius) OR (MH "mekong valley") OR (MH melanesia) OR (MH micronesia) OR (MH monaco) OR (MH mongolia) OR (MH montenegro) OR (MH morocco) OR (MH mozambique) OR (MH myanmar) OR (MH namibia) OR (MH nepal) OR (MH nicaragua) OR (MH niger) OR (MH nigeria) OR (MH oman) OR (MH pakistan) OR (MH palau) OR (MH panama+) OR (MH "papua new guinea") OR (MH paraguay) OR (MH peru) OR (MH philippines) OR (MH qatar) OR (MH "republic of belarus") OR (MH "republic of north macedonia") OR (MH romania) OR (MH russia+) OR (MH rwanda) OR (MH "saint kitts and nevis") OR (MH "saint lucia") OR (MH "saint vincent and the grenadines") OR (MH "sao tome and principe") OR (MH "saudi arabia") OR (MH serbia) OR (MH "sierra leone") OR (MH senegal) OR (MH seychelles) OR (MH singapore) OR (MH somalia) OR (MH "south sudan") OR (MH "sri lanka") OR (MH sudan) OR (MH suriname) OR (MH syria) OR (MH taiwan) OR (MH tajikistan) OR (MH tanzania) OR (MH thailand) OR (MH timor-leste) OR (MH togo) OR (MH tonga) OR (MH "trinidad and tobago") OR (MH tunisia) OR (MH turkmenistan) OR (MH uganda) OR (MH ukraine) OR (MH "united arab emirates") OR (MH uruguay) OR (MH uzbekistan) OR (MH vanuatu) OR (MH venezuela) OR (MH vietnam) OR (MH "west indies") OR (MH yemen) OR (MH zambia) OR (MH zimbabwe) | Limiters - Age Groups: All Adult Expanders - Apply equivalent subjects Search modes - Find all my search terms | 158,197 |
| 18 | OECD OR (MH "European Union") OR (MH "Developed Countries") OR (MH australasia) OR (MH australia+) OR (MH austria) OR (MH "baltic states") OR (MH belgium) OR (MH canada+) OR (MH chile) OR (MH colombia) OR (MH "costa rica") OR (MH "czech republic") OR (MH denmark+) OR (MH estonia) OR (MH europe) OR (MH finland) OR (MH france+) OR (MH germany+) OR (MH greece) OR (MH hungary) OR (MH iceland) OR (MH ireland) OR (MH israel) OR (MH italy+) OR (MH japan+) OR (MH korea) OR (MH latvia) OR (MH lithuania) OR (MH luxembourg) OR (MH mexico) OR (MH netherlands) OR (MH "new zealand") OR (MH "north america") OR (MH norway+) OR (MH poland) OR (MH portugal) OR (MH "republic of korea+") OR (MH "scandinavian and nordic countries") OR (MH slovakia) OR (MH slovenia) OR (MH spain) OR (MH sweden) OR (MH switzerland) OR (MH turkey) OR (MH "united kingdom+") OR (MH "united states+") | Limiters - Age Groups: All Adult Expanders - Apply equivalent subjects Search modes - Find all my search terms | 526,098 |
| 19 | S17 NOT S18 | Limiters - Age Groups: All Adult Expanders - Apply equivalent subjects Search modes - Find all my search terms | 147,720 |
| 20 | S16 NOT S19 | Limiters - Age Groups: All Adult Expanders - Apply equivalent subjects Search modes - Find all my search terms | 147 |
| 21 | S20 | Limiters - Publication Type: Meta Analysis, Meta Synthesis, Systematic Review Expanders - Apply equivalent subjects Search modes - Find all my search terms | 3 |
| 22 | S20 NOT S21 | Expanders - Apply equivalent subjects Search modes - Find all my search terms | 144 |

Innovative Delivery Models Grey Literature Sources Searched

| Source | Website Link |
| --- | --- |
| U.S. Preventive Service Task Force (USPSTF) | <https://www.uspreventiveservicestaskforce.org/uspstf/> |
| Planetree | <https://planetree.org/certification/> |
| Mayo Clinic Shared Decision-Making National Resource Center | <https://www.mayoclinic.org/> |
| Agency for Healthcare Research and Quality (AHRQ) | <https://www.ahrq.gov/> |
| Centers for Disease Control and Prevention (CDC) | <https://www.cdc.gov/> |
| Institute for Patient- and Family-Centered Care | <https://www.ipfcc.org/> |
| Institute for Healthcare Improvement (IHI) | <https://www.ihi.org/> |
| National Academy of Medicine (NAM) | <https://nam.edu/> |
| Centers for Medicare & Medicaid Services (CMS) Innovation Center | <https://innovation.cms.gov/> |
| American College of Physicians (ACP) | <https://www.acponline.org/> |
| National Cancer Institute, Division of Cancer Control & Population Sciences (National Institutes of Health), Practice Tools | <https://cancercontrol.cancer.gov/is/tools/practice-tools> |
| National Institute for Health and Care Excellence | <https://www.nice.org.uk/> |
| Beryl Institute | <https://www.theberylinstitute.org/> |
| The Guide to Community Preventive Services | <https://www.thecommunityguide.org/> |
| The Pathways to Prevention (P2P) Program | <https://prevention.nih.gov/research-priorities/research-needs-and-gaps/pathways-prevention> |
| U.S. Department of Veterans Affairs Evidence Synthesis Program | <https://www.hsrd.research.va.gov/publications/esp/> |
| McMaster Health Systems Evidence | <https://www.healthsystemsevidence.org/> |
| Health Resources & Services Administration, Training and Technical Assistance Hub | <https://www.hrsa.gov/library/performance-measurement-quality-improvement> |
| Health Services: Cost Review Commission (HSCRC) | <https://hscrc.maryland.gov/Pages/default.aspx> |
| American Speech-Language-Hearing Association, Person- and Family-Centered Care | <https://www.asha.org/practice-portal/clinical-topics/aphasia/person-and-family-centered-care/> |
| Evaluating the Nation’s Largest Primary Care Delivery Model: Comprehensive Primary Care Plus (CPC+) | <https://www.mathematica.org/projects/evaluating-the-nations-largest-primary-care-delivery-initiative> |

Innovative Delivery Models Technical Expert Panel Participants and Key Informants

The list of people who participated in the technical expert panel (TEP) or as key informants for the innovative delivery models focus area is provided in Table A-6.

Table A-6. Innovative Delivery Models Technical Expert Panel Members and Key Informants

| Name | Organization | Role | Type of Organization |
| --- | --- | --- | --- |
| Mari-Lynn Drainoni, PhD, Med | Boston University Chobanian & Avedisian School of Medicine | Research Professor | Research/academia |
| C. Annette DuBard, MD, MPH | Aledade | Senior Advisor and Vice President of Clinical Strategy | Other |
| Glyn Elwin, MD, MSc, PhD, FRCGP | The Dartmouth Institute for Health Policy & Clinical Practice | Professor | Research/academia |
| Susan B. Frampton, PhD, FPCC | Planetree International | President | Other |
| Ann Greiner, MCP | Primary Care Collaborative | President and CEO | Other |
| Scott Hammond, MD, FAAFP | Colorado Center for Primary Care Innovation | Clinical Professor of Family Medicine, Co-Chair CO Primary Care Collaborative | AHRQ Primary Care Learning Community |
| Jennifer Haas, MD, MSc | Massachusetts General Hospital | Professor of Medicine, Director of Research | Healthcare system |
| Jane Kim, MD, MPH | Veterans Health Administration, National Center for Health Promotion and Disease Prevention | Executive Director of Preventive Medicine | Federal agency |
| Barbara Kivowitz, MSW | Independent Consultant | Patient and Family Advisor | Patient/consumer representative |
| Carol Mangione, MD, MSPH | UCLA Division of General Internal Medicine and Health Services Research | Distinguished Professor of Medicine and Public Health; Former Chair USPSTF | USPSTF (current/former member) |
| Anthony Olson, PhD, PharmD, Med | Essentia Institute of Rural Health | Research Scientist | AHRQ Primary Care Learning Community |
| Charlene Rothkopf | Wellness Consulting Group | Founder and President | Patient/consumer representative |
| Karla Silverman, RN, CNM, MS | Center for Health Care Strategies | Associate Director, Complex Care Delivery | Other |
| Traci Solt, DNP, FACHE, NEA-BC, AMC-BC, CCM, CRRN | National Veterans Health Administration, Office of Primary Care | Director for Clinical Services | Federal agency |
| Glan Taksler, PhD | Cleveland Clinic | Associate Professor | Research/academia |
| Janice Tufte | Hassanah Consulting | Lived Experience Consultant | Patient/consumer Representative |
| Anthony Viera, MD, MPH | Duke University School of Medicine | Professor and Chair, Department of Family Medicine and Community Health | Healthcare system |
| Scott Young, MD | Kaiser Permanent Care Management Institute | Executive Director, Care Management Institute | Healthcare system |
| Anonymous | - | - | 1 from a Healthcare system |

Innovative Delivery Models: Technical Expert Panel and Key Informant Interview Questions

- What are models/exemplars for providing person-centered preventive care for CPS?
- What approaches could facilitate achieving the delivery of person-centered preventive care by primary care providers, and what may get in the way of using these approaches?
- How can primary care practices be transformed to better support comprehensive person-centered delivery of preventive care services?
- What are additional examples of successful implementation of personalized risk assessment approaches in real world settings? What organizations have an established record in these areas?
- Are there resources, toolkits, or implementation guides related to the use of personalized risk assessment approaches to improve CPS delivery in real-world settings?
- How can personalized risk assessment and follow-up approaches be broadly adapted to advance person-centered delivery of preventive care?
  - Consider anticipated barriers and possible facilitators to real world use
- How can these approaches be further adapted to help deliver comprehensive person-centered preventive care?
- What are the next steps to advance provision of comprehensive person-centered delivery of clinical preventive services within healthcare systems? What funding strategies are needed to advance in this area?
- What are areas where more research is needed? What type of models or approaches are ready for large scale dissemination and demonstration projects?
- Any other comments or suggestions for next steps? Do you have recommendations for other experts in this area that we should speak with?

Public Health and Community Linkages Environmental Scan Data Sources and Searches

Searches of electronic databases were limited to PubMed, CINAHL, and the Cochrane Library (**Table A-7**). The searches covered the period from January 1, 2012, to February 7, 2023. We did not limit our search to specific types of CPS. We also searched gray literature sources using variations of search terms related to primary care, public health, prevention, screening, and collaborate. In addition to electronic databases and websites, we manually searched reference lists from relevant review articles. All citations were managed and deduplicated using EndNote X9 (Clarivate Analytics). DistillerSR was used to manage the abstract and article screening and review process.

**Table A-7**. Search Strategy for the Public Health Linkages Topic Area

| Search Number | PubMed Query | Filters | Results |
| --- | --- | --- | --- |
| 1 | ("Primary Health Care"[Majr] OR "Physicians, Primary Care"[Majr] OR "primary care"[title] OR "primary health care"[title] OR PHC[title] OR "General Practice"[Majr] OR "Family Practice"[Majr] OR "general practice"[title] OR "family practice"[title] OR "Preventive Health Services"[Majr] OR "Preventive Medicine"[Majr] OR "General Practice"[Majr] OR "general practice"[title] OR "family practice"[title] OR "Preventive Health Services"[Majr] OR "Preventive Medicine"[Majr] OR "Community Health Centers"[Majr] OR "Federally Qualified Health Center"[title] OR "Federally Qualified Health Centers"[title] OR FQHC*[title]) |  | [569,947](https://pubmed.ncbi.nlm.nih.gov/?term=%28%22Primary+Health+Care%22%5BMajr%5D+OR+%22Physicians%2C+Primary+Care%22%5BMajr%5D+OR+%22primary+care%22%5Btitle%5D+OR+%22primary+health+care%22%5Btitle%5D+OR+PHC%5Btitle%5D+OR+%22General+Practice%22%5BMajr%5D+OR+%22Family+Practice%22%5BMajr%5D+OR+%22general+practice%22%5Btitle%5D+OR+%22family+practice%22%5Btitle%5D+OR+%22Preventive+Health+Services%22%5BMajr%5D+OR+%22Preventive+Medicine%22%5BMajr%5D+OR+%22General+Practice%22%5BMajr%5D+OR+%22general+practice%22%5Btitle%5D+OR+%22family+practice%22%5Btitle%5D+OR+%22Preventive+Health+Services%22%5BMajr%5D+OR+%22Preventive+Medicine%22%5BMajr%5D+OR+%22Community+Health+Centers%22%5BMajr%5D+OR+%22Federally+Qualified+Health+Center%22%5Btitle%5D+OR+%22Federally+Qualified+Health+Centers%22%5Btitle%5D+OR+FQHC%2A%5Btitle%5D%29&sort=relevance&size=50&ac=no) |
| 2 | "Public Health"[Majr] OR "Public Health Administration"[Majr] OR "Public Health Nursing"[Majr] OR "Public Health Surveillance"[Majr] OR "public health"[title] OR "Population Health"[Majr] OR "population health"[title] |  | [2,105,736](https://pubmed.ncbi.nlm.nih.gov/?term=%22Public+Health%22%5BMajr%5D+OR+%22Public+Health+Administration%22%5BMajr%5D+OR+%22Public+Health+Nursing%22%5BMajr%5D+OR+%22Public+Health+Surveillance%22%5BMajr%5D+OR+%22public+health%22%5Btitle%5D+OR+%22Population+Health%22%5BMajr%5D+OR+%22population+health%22%5Btitle%5D&sort=relevance&size=50&ac=no) |
| 3 | #1 AND #2 |  | [219,757](https://pubmed.ncbi.nlm.nih.gov/?term=%231+AND+%232&sort=relevance&size=50&ac=no) |
| 4 | "Community based organization*"[tiab] OR church*[title] OR "Faith-Based Organizations"[Majr] OR "community engagement"[title] OR "community-based organisation"[title] OR "community based organisations"[title] OR "Community-Based Participatory Research"[Majr] OR "community-based participatory research"[title] OR "Community-based program"[title] OR "community-based programs"[title] OR CBPR[tiab] OR "Clinic-community linkages"[tiab] |  | [10,750](https://pubmed.ncbi.nlm.nih.gov/?term=%22Community+based+organization%2A%22%5Btiab%5D+OR+church%2A%5Btitle%5D+OR+%22Faith-Based+Organizations%22%5BMajr%5D+OR+%22community+engagement%22%5Btitle%5D+OR+%22community-based+organisation%22%5Btitle%5D+OR+%22community+based+organisations%22%5Btitle%5D+OR+%22Community-Based+Participatory+Research%22%5BMajr%5D+OR+%22community-based+participatory+research%22%5Btitle%5D+OR+%22Community-based+program%22%5Btitle%5D+OR+%22community-based+programs%22%5Btitle%5D+OR+CBPR%5Btiab%5D+OR+%22Clinic-community+linkages%22%5Btiab%5D&sort=relevance&size=50&ac=no) |
| 5 | #1 AND #4 |  | [1,793](https://pubmed.ncbi.nlm.nih.gov/?term=%231+AND+%234&sort=relevance&size=50&ac=no) |
| 6 | #3 OR #5 |  | [221,073](https://pubmed.ncbi.nlm.nih.gov/?term=%233+OR+%235&sort=relevance&size=50&ac=no) |
| 7 | "Models, Organizational"[Majr] OR "Organizational Innovation"[Majr] OR Collaboration[title] OR "Health Facility Merger"[Majr] OR "Systems Integration"[Majr] OR "Cooperative Behavior"[Majr] OR Integration[title] OR integrated[title] OR "organizational model*"[title] OR "collaborative"[title] OR Partnership[title] OR partner[title] OR partners[title] OR linkage*[title/abstract] OR cooperation[title] OR integration[title] |  | [344,809](https://pubmed.ncbi.nlm.nih.gov/?term=%22Models%2C+Organizational%22%5BMajr%5D+OR+%22Organizational+Innovation%22%5BMajr%5D+OR+Collaboration%5Btitle%5D+OR+%22Health+Facility+Merger%22%5BMajr%5D+OR+%22Systems+Integration%22%5BMajr%5D+OR+%22Cooperative+Behavior%22%5BMajr%5D+OR+Integration%5Btitle%5D+OR+integrated%5Btitle%5D+OR+%22organizational+model%2A%22%5Btitle%5D+OR+%22collaborative%22%5Btitle%5D+OR+Partnership%5Btitle%5D+OR+partner%5Btitle%5D+OR+partners%5Btitle%5D+OR+linkage%2A%5Btitle%2Fabstract%5D+OR+cooperation%5Btitle%5D+OR+integration%5Btitle%5D&sort=relevance&size=50&ac=no) |
| 8 | #6 AND #7 |  | [3,924](https://pubmed.ncbi.nlm.nih.gov/?term=%236+AND+%237&sort=relevance&size=50&ac=no) |
| 9 | "Delivery of Health Care"[Majr] OR "Delivery of Health Care, Integrated"[Majr] OR "Patient Care Team"[Majr] OR "Continuity of Patient Care"[Majr] OR "Patient-Centered Care"[Mesh] OR "Patient Centered"[title] OR "Person centered"[title] OR "Care coordination"[title:~2] OR "coordinated care"[title:~2] |  | [921,237](https://pubmed.ncbi.nlm.nih.gov/?term=%22Delivery+of+Health+Care%22%5BMajr%5D+OR+%22Delivery+of+Health+Care%2C+Integrated%22%5BMajr%5D+OR+%22Patient+Care+Team%22%5BMajr%5D+OR+%22Continuity+of+Patient+Care%22%5BMajr%5D+OR+%22Patient-Centered+Care%22%5BMesh%5D+OR+%22Patient+Centered%22%5Btitle%5D+OR+%22Person+centered%22%5Btitle%5D+OR+%22Care+coordination%22%5Btitle%3A~2%5D+OR+%22coordinated+care%22%5Btitle%3A~2%5D&sort=relevance&size=50&ac=no) |
| 10 | #8 AND #9 |  | [1,137](https://pubmed.ncbi.nlm.nih.gov/?term=%238+AND+%239&sort=relevance&size=50&ac=no) |
| 11 | "Preventive Health Services"[Mesh] OR "clinical preventive services"[tiab] OR "clinical preventive service"[tiab] OR Preventive[title] OR Prevention[title] OR Mass Screening[Mesh] OR screening[title] OR screen*[title] OR screens[title] OR screened[title] OR Counseling[Mesh] OR counsel*[title] OR counseling[title] OR Immunization[Mesh] OR Vaccination[Mesh] OR "Immunization Programs"[Mesh] OR immunization[title] OR vaccine*[title] OR vaccination*[title] OR "Primary Prevention"[Majr] OR "Chronic Disease/prevention and control"[Mesh] |  | [1,211,923](https://pubmed.ncbi.nlm.nih.gov/?term=%22Preventive+Health+Services%22%5BMesh%5D+OR+%22clinical+preventive+services%22%5Btiab%5D+OR+%22clinical+preventive+service%22%5Btiab%5D+OR+Preventive%5Btitle%5D+OR+Prevention%5Btitle%5D+OR+Mass+Screening%5BMesh%5D+OR+screening%5Btitle%5D+OR+screen%2A%5Btitle%5D+OR+screens%5Btitle%5D+OR+screened%5Btitle%5D+OR+Counseling%5BMesh%5D+OR+counsel%2A%5Btitle%5D+OR+counseling%5Btitle%5D+OR+Immunization%5BMesh%5D+OR+Vaccination%5BMesh%5D+OR+%22Immunization+Programs%22%5BMesh%5D+OR+immunization%5Btitle%5D+OR+vaccine%2A%5Btitle%5D+OR+vaccination%2A%5Btitle%5D+OR+%22Primary+Prevention%22%5BMajr%5D+OR+%22Chronic+Disease%2Fprevention+and+control%22%5BMesh%5D&sort=relevance&size=50&ac=no) |
| 12 | #8 AND #11 |  | [2,933](https://pubmed.ncbi.nlm.nih.gov/?term=%238+AND+%2311&sort=relevance&size=50&ac=no) |
| 13 | #10 OR #12 |  | [3,412](https://pubmed.ncbi.nlm.nih.gov/?term=%2310+OR+%2312&sort=relevance&size=50&ac=no) |
| 14 | Intervention*[tw] OR program[tiab] OR programs[tiab] OR "Evaluation Study"[PT] OR "Evaluation Studies as Topic"[Mesh] OR "Program Evaluation"[Mesh] OR Evaluation[ti] OR "Health Plan Implementation"[Mesh] OR "Health Impact Assessment"[Mesh] OR "Patient Outcome Assessment"[Mesh] |  | [3,630,275](https://pubmed.ncbi.nlm.nih.gov/?term=Intervention%2A%5Btw%5D+OR+program%5Btiab%5D+OR+programs%5Btiab%5D+OR+%22Evaluation+Study%22%5BPT%5D+OR+%22Evaluation+Studies+as+Topic%22%5BMesh%5D+OR+%22Program+Evaluation%22%5BMesh%5D+OR+Evaluation%5Bti%5D+OR+%22Health+Plan+Implementation%22%5BMesh%5D+OR+%22Health+Impact+Assessment%22%5BMesh%5D+OR+%22Patient+Outcome+Assessment%22%5BMesh%5D&sort=relevance&size=50&ac=no) |
| 15 | #13 AND #14 |  | [1,739](https://pubmed.ncbi.nlm.nih.gov/?term=%2313+AND+%2314&sort=relevance&size=50&ac=no) |
| 16 | #13 AND #14 | English | [1,662](https://pubmed.ncbi.nlm.nih.gov/?term=%2313+AND+%2314&filter=lang.english&ac=no&size=50&sort=relevance) |
| 17 | #13 AND #14 | English, from 2012 - 2023 | [1,092](https://pubmed.ncbi.nlm.nih.gov/?term=%2313+AND+%2314&filter=lang.english&filter=years.2012-2023&ac=no&size=50&sort=relevance) |
| 18 | ("Animals"[Mesh] NOT "Humans"[Mesh]) OR rats[tw] OR cow[tw] OR cows[tw] OR chicken[tw] OR chickens[tw] OR horse[tw] OR horses[tw] OR mice[tw] OR mouse[tw] OR bovine[tw] OR sheep[tw] OR ovine[tw] OR murine[tw] OR murinae[tw] |  | [6,379,783](https://pubmed.ncbi.nlm.nih.gov/?term=%28%22Animals%22%5BMesh%5D+NOT+%22Humans%22%5BMesh%5D%29+OR+rats%5Btw%5D+OR+cow%5Btw%5D+OR+cows%5Btw%5D+OR+chicken%5Btw%5D+OR+chickens%5Btw%5D+OR+horse%5Btw%5D+OR+horses%5Btw%5D+OR+mice%5Btw%5D+OR+mouse%5Btw%5D+OR+bovine%5Btw%5D+OR+sheep%5Btw%5D+OR+ovine%5Btw%5D+OR+murine%5Btw%5D+OR+murinae%5Btw%5D&sort=relevance&size=50&ac=no) |
| 19 | #17 NOT #18 |  | [1,085](https://pubmed.ncbi.nlm.nih.gov/?term=%2317+NOT+%2318&sort=relevance&size=50&ac=no) |
| 20 | #17 NOT #18 | Systematic Review | [31](https://pubmed.ncbi.nlm.nih.gov/?term=%2317+NOT+%2318&filter=pubt.systematicreview&ac=no&size=50&sort=relevance) |
| 21 | #17 NOT #18 | Meta-Analysis, Systematic Review | [35](https://pubmed.ncbi.nlm.nih.gov/?term=%2317+NOT+%2318&filter=pubt.meta-analysis&filter=pubt.systematicreview&ac=no&size=50&sort=relevance) |
| 22 | #19 AND ("scoping review"[tiab] OR "integrative review"[tiab] OR "rapid review"[tiab] OR "living review"[tiab] OR "environmental scan"[tiab]) |  | [14](https://pubmed.ncbi.nlm.nih.gov/?term=%2319+AND+%28%22scoping+review%22%5Btiab%5D+OR+%22integrative+review%22%5Btiab%5D+OR+%22rapid+review%22%5Btiab%5D+OR+%22living+review%22%5Btiab%5D+OR+%22environmental+scan%22%5Btiab%5D%29&sort=relevance&size=50&ac=no) |
| 23 | #22 NOT #21 |  | [11](https://pubmed.ncbi.nlm.nih.gov/?term=%2322+NOT+%2321&sort=relevance&size=50&ac=no) |
| 24 | #19 NOT (#21 OR #23) |  | [1,039](https://pubmed.ncbi.nlm.nih.gov/?term=%2319+NOT+%28%2321+OR+%2323%29&sort=relevance&size=50&ac=no) |
| 25 | toolkit[tw] OR toolkits[tw] OR "tool kit"[tw] OR "tool kits"[tw] |  | [11,429](https://pubmed.ncbi.nlm.nih.gov/?term=toolkit%5Btw%5D+OR+toolkits%5Btw%5D+OR+%22tool+kit%22%5Btw%5D+OR+%22tool+kits%22%5Btw%5D&sort=relevance&size=50&ac=no) |
| 26 | #19 AND #25 |  | [4](https://pubmed.ncbi.nlm.nih.gov/?term=%2319+AND+%2325&sort=relevance&size=50&ac=no) |

| Search Number | Cochrane Library Query | Filters | Results |
| --- | --- | --- | --- |
| 1 | ([mh "Primary Health Care"] OR [mh "Physicians, Primary Care"] OR "primary care":ti OR "primary health care":ti OR PHC:ti OR [mh "General Practice"] OR [mh "Family Practice"] OR "general practice":ti OR "family practice":ti OR [mh "Preventive Health Services"] OR [mh "Preventive Medicine"] OR [mh "General Practice"] OR "general practice":ti OR "family practice":ti OR [mh "Preventive Health Services"] OR [mh "Preventive Medicine"] OR [mh "Community Health Centers"] OR "Federally Qualified Health Center":ti OR "Federally Qualified Health Centers":ti OR FQHC*:ti) |  | 55,876 |
| 2 | [mh "Public Health"] OR [mh "Public Health Administration"] OR [mh "Public Health Nursing"] OR [mh "Public Health Surveillance"] OR "public health":ti OR [mh "Population Health"] OR "population health":ti |  | 551,201 |
| 3 | #1 AND #2 |  | 41,430 |
| 4 | (" "Community based" NEXT organization*"):ti,ab OR church*:ti OR [mh "Faith-Based Organizations"] OR "community engagement":ti OR "community-based organisation":ti OR "community based organisations":ti OR [mh "Community-Based Participatory Research"] OR "community-based participatory research":ti OR "Community-based program":ti OR "community-based programs":ti OR CBPR:ti,ab OR "Clinic-community linkages":ti,ab |  | 41,071 |
| 5 | #1 AND #4 |  | 4,664 |
| 6 | #3 OR #5 |  | 42,589 |
| 7 | [mh "Models, Organizational"] OR [mh "Organizational Innovation"] OR Collaboration:ti OR [mh "Health Facility Merger"] OR [mh "Systems Integration"] OR [mh "Cooperative Behavior"] OR Integration:ti OR integrated:ti OR ("organizational" NEXT model*):ti OR collaborative:ti OR Partnership:ti OR partner:ti OR partners:ti OR linkage*:ti,ab OR cooperation:ti OR integration:ti |  | 15,692 |
| 8 | #6 AND #7 |  | 1,418 |
| 9 | [mh "Delivery of Health Care"] OR [mh "Delivery of Health Care, Integrated"] OR [mh "Patient Care Team"] OR [mh "Continuity of Patient Care"] OR [mh "Patient-Centered Care"] OR "Patient Centered":ti OR "Person centered":ti OR "Care coordination":ti |  | 88,238 |
| 10 | #8 AND #9 |  | 765 |
| 11 | [mh "Preventive Health Services"] OR "clinical preventive services":ti,ab OR "clinical preventive service":ti,ab OR Preventive:ti OR Prevention:ti OR [mh "Mass Screening"] OR screening:ti OR screen*:ti OR screens:ti OR screened:ti OR [mh Counseling] OR counsel*:ti OR counseling:ti OR [mh Immunization] OR [mh Vaccination] OR [mh "Immunization Programs"] OR immunization:ti OR vaccine*:ti OR vaccination*:ti OR [mh "Primary Prevention"] OR [mh "Chronic Disease"] |  | 154,897 |
| 12 | #8 AND #11 |  | 952 |
| 13 | #10 OR #12 |  | 1,275 |
| 14 | Intervention*:ti,ab,kw OR program:ti,ab OR programs:ti,ab OR "Evaluation Study":pt OR [mh "Evaluation Studies as Topic"] OR [mh "Program Evaluation"] OR Evaluation:ti OR [mh "Health Plan Implementation"] OR [mh "Health Impact Assessment"] OR [mh "Patient Outcome Assessment"] |  | 658,798 |
| 15 | #13 AND #14 |  | 1,052 |
| 16 | ([mh Animals] NOT [mh Humans]) OR rats:ti,ab,kw OR cow:ti,ab,kw OR cows:ti,ab,kw OR chicken:ti,ab,kw OR chickens:ti,ab,kw OR horse:ti,ab,kw OR horses:ti,ab,kw OR mice:ti,ab,kw OR mouse:ti,ab,kw OR bovine:ti,ab,kw OR sheep:ti,ab,kw OR ovine:ti,ab,kw OR murine:ti,ab,kw OR murinae:ti,ab,kw |  | 18,476 |
| 17 | #15 NOT #16 |  | 1,049 |
| 18 | #17 | Limited to Cochrane Reviews published 2012-2023 | 6 |
| 19 | #17 | Limited to Trials published 2012-2023 | 674 |
| 20 | toolkit:ti,ab,kw OR toolkits:ti,ab,kw OR "tool kit":ti,ab,kw OR "tool kits":ti,ab,kw |  | 776 |
| 21 | #17 AND #20 (all are Trials) | Limited to Trials published 2012-2023 | 5 |

| Search Number | CINAHL Query | Limiters/Expanders | Results |
| --- | --- | --- | --- |
| 1 | ((MM "Primary Health Care+") OR (MM "Physicians, Primary Care+") OR (TI "primary care") OR (TI "primary health care") OR (TI PHC) OR (MM "General Practice+") OR (MM "Family Practice+") OR (TI "general practice") OR (TI "family practice") OR (MM "Preventive Health Services+") OR (MM "Preventive Medicine+") OR (MM "General Practice+") OR (TI "general practice") OR (TI "family practice") OR (MM "Preventive Health Services+") OR (MM "Preventive Medicine+") OR (MM "Community Health Centers+") OR (TI "Federally Qualified Health Center") OR (TI "Federally Qualified Health Centers") OR (TI FQHC*)) | Expanders - Apply equivalent subjects Search modes - Find all my search terms | 83,977 |
| 2 | (MM "Public Health+") OR (MM "Public Health Administration+") OR (MM "Public Health Nursing+") OR (MM "Public Health Surveillance+") OR (TI "public health") OR (MM "Population Health+") OR (TI "population health") | Expanders - Apply equivalent subjects Search modes - Find all my search terms | 578,450 |
| 3 | S1 AND S2 | Expanders - Apply equivalent subjects Search modes - Find all my search terms | 7,808 |
| 4 | (TI "Community based organization*" OR AB "Community based organization*") OR (TI church*) OR (MM "Faith-Based Organizations+") OR (TI "community engagement") OR (TI "community-based organisation") OR (TI "community-based organisations") OR (MM "Community-Based Participatory Research+") OR (TI "community-based participatory research") OR (TI "Community-based program") OR (TI "community-based programs") OR (TI CBPR OR AB CBPR) OR (TI "Clinic-community linkages" OR AB "Clinic-community linkages") | Expanders - Apply equivalent subjects Search modes - Find all my search terms | 4,890 |
| 5 | S1 AND S4 | Expanders - Apply equivalent subjects Search modes - Find all my search terms | 90 |
| 6 | S3 OR S5 | Expanders - Apply equivalent subjects Search modes - Find all my search terms | 7,885 |
| 7 | (MM "Models, Organizational+") OR (MM "Organizational Innovation+") OR (TI Collaboration) OR (MM "Health Facility Merger+") OR (MM "Systems Integration+") OR (MM "Cooperative Behavior+") OR (TI Integration) OR (TI integrated) OR (TI "organizational model*") OR (TI collaborative) OR (TI Partnership) OR (TI partner) OR (TI partners) OR (TI linkage* OR AB linkage*) OR (TI cooperation) OR (TI integration) | Expanders - Apply equivalent subjects Search modes - Find all my search terms | 102,338 |
| 8 | S6 AND S7 | Expanders - Apply equivalent subjects Search modes - Find all my search terms | 347 |
| 9 | (MM "Delivery of Health Care+") OR (MM "Delivery of Health Care, Integrated+") OR (MM "Patient Care Team+") OR (MM "Continuity of Patient Care+") OR (MH "Patient-Centered Care+") OR (TI "Patient Centered") OR (TI "Person centered") OR (TI "Care coordination") OR (TI "coordinated care") | Expanders - Apply equivalent subjects Search modes - Find all my search terms | 17,504 |
| 10 | S8 AND S9 | Expanders - Apply equivalent subjects Search modes - Find all my search terms | 9 |
| 11 | (MH " "Preventive Health Services"+") OR (TI "clinical preventive services" OR AB "clinical preventive services") OR (TI "clinical preventive service" OR AB "clinical preventive service") OR (TI Preventive) OR (TI Prevention) OR (MH "Mass Screening+") OR (TI screening) OR (TI screen*) OR (TI screens) OR (TI screened) OR (MH Counseling+) OR (TI counsel*) OR (TI counseling) OR (MH Immunization+) OR (MH Vaccination+) OR (MH "Immunization Programs+") OR (TI immunization) OR (TI vaccine*) OR (TI vaccination*) OR (MM "Primary Prevention+") OR (MH "Chronic Disease/prevention and control+") | Expanders - Apply equivalent subjects Search modes - Find all my search terms | 338,523 |
| 12 | S8 AND S11 | Expanders - Apply equivalent subjects Search modes - Find all my search terms | 116 |
| 13 | S10 OR S12 | Expanders - Apply equivalent subjects Search modes - Find all my search terms | 124 |
| 14 | Intervention* OR (TI program OR AB program) OR (TI programs OR AB programs) OR (PT "Evaluation Study") OR (MH "Evaluation Studies as Topic+") OR (MH "Program Evaluation+") OR (TI Evaluation) OR (MH "Health Plan Implementation+") OR (MH "Health Impact Assessment+") OR (MH "Patient Outcome Assessment+") | Expanders - Apply equivalent subjects Search modes - Find all my search terms | 1,004,937 |
| 15 | S13 AND S14 | Expanders - Apply equivalent subjects Search modes - Find all my search terms | 56 |
| 16 | ((MH Animals+) NOT (MH Humans+)) OR rats OR cow OR cows OR chicken OR chickens OR horse OR horses OR mice OR mouse OR bovine OR sheep OR ovine OR murine OR murinae | Expanders - Apply equivalent subjects Search modes - Find all my search terms | 258,843 |
| 17 | S15 NOT S16 | Expanders - Apply equivalent subjects Search modes - Find all my search terms | 56 |
| 18 | S17 | Limiters - Published Date: 20120101-20231231; English Language; Language: English Expanders - Apply equivalent subjects Search modes - Find all my search terms | 42 |
| 19 | S18 | Limiters - Publication Type: Anecdote, Case Study Expanders - Apply equivalent subjects Search modes - Find all my search terms | 1 |
| 20 | S18 | Limiters - Publication Type: Meta Analysis, Meta Synthesis, Systematic Review Expanders - Apply equivalent subjects Search modes - Find all my search terms | 1 |
| 21 | S18 AND ((TI "scoping review" OR AB "scoping review") OR (TI "integrative review" OR AB "integrative review") OR (TI "rapid review" OR AB "rapid review") OR (TI "living review" OR AB "living review") OR (TI "environmental scan" OR AB "environmental scan")) | Expanders - Apply equivalent subjects Search modes - Find all my search terms | 1 |
| 22 | S21 NOT S20 | Expanders - Apply equivalent subjects Search modes - Find all my search terms | 0 |
| 23 | S18 AND (toolkit" OR toolkits OR "tool kit" OR "tool kits") | Expanders - Apply equivalent subjects Search modes - Find all my search terms | 0 |
| 24 | S18 NOT (S19 OR S20 OR S21) | Expanders - Apply equivalent subjects Search modes - Find all my search terms | 40 |

Public Health Linkages Grey Literature Sources Searched

| Source | Website Link |
| --- | --- |
| Google and Google Scholar | <https://www.google.com/> and <https://scholar.google.com/> |
| AHRQ: Improving Primary Care Practice | <https://www.ahrq.gov/ncepcr/tools/improve/index.html> |
| AHRQ: Primary Care Practice-Based Research Networks | <https://www.ahrq.gov/research/findings/factsheets/primary/pbrn/index.html> |
| AHRQ: Health Care Innovation Exchange | <https://www.ahrq.gov/innovations/index.html> |
| American College of Physicians | <https://www.acponline.org/> |
| American Academy of Pediatrics | <https://www.aap.org/> |
| American Academy of Family Physicians | <https://www.aafp.org/home.html> |
| Centers for Disease Control and Prevention | <https://www.cdc.gov/> |
| Community Preventive Task Force: The Community Guide | <https://www.thecommunityguide.org/> |
| U.S. Preventive Services Task Force | <https://www.uspreventiveservicestaskforce.org/uspstf/> |
| American Public Health Association | <https://www.apha.org/> |
| National Association of Country and City Health Officials | <https://www.naccho.org/> |
| National Association of Community Health Centers | <http://www.nachc.org> |
| de Beaumont Foundation | <https://debeaumont.org/programs/> |
| Robert Wood Johnson Foundation | <https://www.rwjf.org/> |
| The Pathways to Prevention (P2P) Program | <https://prevention.nih.gov/research-priorities/research-needs-and-gaps/pathways-prevention> |
| Joanna Briggs Institute | <https://jbi.global/> |
| U.S. Department of Veterans Affairs Evidence Synthesis Program | <https://www.hsrd.research.va.gov/publications/esp/> |
| McMaster University Health System Evidence | <https://library.mcmaster.ca/databases/health-systems-evidence> |
| National Institute for Health and Care Excellence | <https://www.nice.org.uk/> |

Public Health Linkages Technical Expert Panel Participants and Key Informants

The list of people who participated in the technical expert panel (TEP) or as key informants for the public health and community linkages focus area is provided in Table A-8.

Table A-8. Public Health and Community Linkages Technical Expert Panel Members and Key Informants

| Name | Organization | Role | Type of Organization |
| --- | --- | --- | --- |
| Toni Eyssallene, MD, PhD | New York City Department of Health and Mental Hygiene | Senior Medical Advisor to the Chief Medical Officer | State policy/public health |
| Karen Hacker, MD, MPH | Centers for Disease Control and Prevention | Director, National Center for Chronic Disease, Prevention and Health Promotion | Federal agency |
| Howard Haft, MD, MMM, CPE, FACPE | American Heart Association Ambulatory Quality Committee, formerly with the Maryland Department of Health | Senior Advisor | State policy/public health |
| Heather Hodge, MEd | YMCA of the USA | Senior Director, Community Health | Other |
| Mitchell Katz, MD | New York City Health + Hospitals | Chief Executive Officer | Healthcare system |
| Kathryn G. Kietzman, PhD, MSW | University of California, Los Angeles, Center for Health Policy Research | Director, Health Equity Program | Research/academia |
| James Macrae, MA, MPP | Health Resources and Services Administration | Associate Administrator for Bureau of Primary Health Care | Federal agency |
| J. Lloyd Michener, MD | Duke University School of Medicine | Professor Emeritus, Department of Family Medicine & Community Health | Research/academia |
| Deborah Porterfield, MD, MPH | Formerly, Department of Family Medicine, UNC-Chapel Hill, and RTI International; currently, Office of the Assistant Secretary for Planning and Evaluation | Medical Officer | Federal agency |
| Shailey Prasad, MD, MPH | University of Minnesota Center for Global Health and Social Responsibility; Department of Family Medicine and Community Health | Executive Director; Vice Chair for Education | Research/academia |
| Melanie A. Reese, SME | Older Women Embracing Life | Executive Director | Patient/consumer representative |
| Alexis Snyder, BA | Independent consultant | Patient and stakeholder engagement specialist | Patient/consumer representative |
| Chelsy Winters, MS | YMCA of Greater Indianapolis | Executive Director of Healthy Living | Other |
| Janet S. Wright, MD, MACC, FPCNA | Centers for Disease Control and Prevention | Director, Division for Heart Disease and Stroke Prevention | Federal agency |
| Anonymous | - | - | 1 from USPSTF (current/former member)  1 from Research/academia  1 from other type of organization |

Public Health Linkages: Technical Expert Panel and Key Informant Interview Questions

- What strategies can be used to strengthen linkages between primary care and public health or community-based organizations to improve delivery of person-centered clinical preventive services?
- How might we improve referral-based programs to:
  - Close the referral loop?
  - Support and sustain community-based programs?
- How might we strengthen ties to primary care with established, evidence-based programs delivered in the community – such as diabetes prevention programs?
  - What resources are needed to strengthen these linkages?
- Reflecting on high priority CPS, where are the opportunities to improve bundled delivery of CPS through clinical community linkages?
- How might we build upon established, disease specific screening and referral programs (e.g., HIV, DPP) to expand to other CPS?
- How might we leverage existing infrastructure and tools for improved data sharing to support delivery of CPS?
- How might we scale examples of co-location of services?
- What are the next steps to advance linkages between primary care and public health or community-based organizations for the delivery of person-centered clinical preventive services?

Disparities in Clinical Preventive Services Environmental Scan Data Sources and Searches

The search for this scan was developed in collaboration with an information specialist based on keywords related to “clinical preventive services”, “equity”, and “disparities” within two bibliographic databases: PubMed and PsychInfo (**Table A-9**). The information specialist focused on two primary sets of literature: (1) synthesized literature (e.g., systematic and narrative reviews) published over the last 10 years and (2) randomized trials published between 2019 and 2023 to build upon a previous systematic review to support the Pathways to Prevention (P2P) Workshop on Achieving Health Equity in Preventive Services.

**Table A-9**. Search Strategy for the Disparities Topic Area

| **Search Number** | **PubMed Query** | **Results** |
| --- | --- | --- |
| 1 | "Preventive Health Services"[Majr] OR "clinical preventive services"[tiab] OR "clinical preventive service"[tiab] OR Preventive[title] OR Prevention[title] OR Mass Screening[Majr] OR screening[title] OR screen*[title] OR screens[title] OR screened[title] OR Counseling[Majr] OR counsel*[title] OR counseling[title] OR Immunization[Majr] OR Vaccination[Majr] OR "Immunization Programs"[Majr] OR immunization[title] OR vaccine*[title] OR vaccination*[title] OR "Primary Prevention"[Majr] | [929,204](https://pubmed.ncbi.nlm.nih.gov/?term=%22Preventive+Health+Services%22%5BMajr%5D+OR+%22clinical+preventive+services%22%5Btiab%5D+OR+%22clinical+preventive+service%22%5Btiab%5D+OR+Preventive%5Btitle%5D+OR+Prevention%5Btitle%5D+OR+Mass+Screening%5BMajr%5D+OR+screening%5Btitle%5D+OR+screen%2A%5Btitle%5D+OR+screens%5Btitle%5D+OR+screened%5Btitle%5D+OR+Counseling%5BMajr%5D+OR+counsel%2A%5Btitle%5D+OR+counseling%5Btitle%5D+OR+Immunization%5BMajr%5D+OR+Vaccination%5BMajr%5D+OR+%22Immunization+Programs%22%5BMajr%5D+OR+immunization%5Btitle%5D+OR+vaccine%2A%5Btitle%5D+OR+vaccination%2A%5Btitle%5D+OR+%22Primary+Prevention%22%5BMajr%5D&sort=relevance&size=100&ac=no) |
| 2 | "Preventive Health Services"[Majr] OR "clinical preventive services"[tiab] OR "clinical preventive service"[tiab] OR Preventive[title] OR Prevention[title] OR Mass Screening[Majr] OR screening[title] OR screen*[title] OR screens[title] OR screened[title] OR Counseling[Majr] OR counsel*[title] OR counseling[title] OR Immunization[Majr] OR Vaccination[Majr] OR "Immunization Programs"[Majr] OR immunization[title] OR vaccine*[title] OR vaccination*[title] OR "Primary Prevention"[Majr] Filters: English | [801,094](https://pubmed.ncbi.nlm.nih.gov/?term=%22Preventive+Health+Services%22%5BMajr%5D+OR+%22clinical+preventive+services%22%5Btiab%5D+OR+%22clinical+preventive+service%22%5Btiab%5D+OR+Preventive%5Btitle%5D+OR+Prevention%5Btitle%5D+OR+Mass+Screening%5BMajr%5D+OR+screening%5Btitle%5D+OR+screen%2A%5Btitle%5D+OR+screens%5Btitle%5D+OR+screened%5Btitle%5D+OR+Counseling%5BMajr%5D+OR+counsel%2A%5Btitle%5D+OR+counseling%5Btitle%5D+OR+Immunization%5BMajr%5D+OR+Vaccination%5BMajr%5D+OR+%22Immunization+Programs%22%5BMajr%5D+OR+immunization%5Btitle%5D+OR+vaccine%2A%5Btitle%5D+OR+vaccination%2A%5Btitle%5D+OR+%22Primary+Prevention%22%5BMajr%5D&filter=lang.english&ac=no&size=100&sort=relevance) |
| 3 | "Preventive Health Services"[Majr] OR "clinical preventive services"[tiab] OR "clinical preventive service"[tiab] OR Preventive[title] OR Prevention[title] OR Mass Screening[Majr] OR screening[title] OR screen*[title] OR screens[title] OR screened[title] OR Counseling[Majr] OR counsel*[title] OR counseling[title] OR Immunization[Majr] OR Vaccination[Majr] OR "Immunization Programs"[Majr] OR immunization[title] OR vaccine*[title] OR vaccination*[title] OR "Primary Prevention"[Majr] Filters: English, from 2012 - 2023 | [388,820](https://pubmed.ncbi.nlm.nih.gov/?term=%22Preventive+Health+Services%22%5BMajr%5D+OR+%22clinical+preventive+services%22%5Btiab%5D+OR+%22clinical+preventive+service%22%5Btiab%5D+OR+Preventive%5Btitle%5D+OR+Prevention%5Btitle%5D+OR+Mass+Screening%5BMajr%5D+OR+screening%5Btitle%5D+OR+screen%2A%5Btitle%5D+OR+screens%5Btitle%5D+OR+screened%5Btitle%5D+OR+Counseling%5BMajr%5D+OR+counsel%2A%5Btitle%5D+OR+counseling%5Btitle%5D+OR+Immunization%5BMajr%5D+OR+Vaccination%5BMajr%5D+OR+%22Immunization+Programs%22%5BMajr%5D+OR+immunization%5Btitle%5D+OR+vaccine%2A%5Btitle%5D+OR+vaccination%2A%5Btitle%5D+OR+%22Primary+Prevention%22%5BMajr%5D&filter=lang.english&filter=years.2012-2023&ac=no&size=100&sort=relevance) |
| 4 | #3 NOT (("Adolescent"[Mesh] OR "Child"[Mesh] OR "Infant"[Mesh]) NOT "Adult"[Mesh]) | [346,642](https://pubmed.ncbi.nlm.nih.gov/?term=%233+NOT+%28%28%22Adolescent%22%5BMesh%5D+OR+%22Child%22%5BMesh%5D+OR+%22Infant%22%5BMesh%5D%29+NOT+%22Adult%22%5BMesh%5D%29&sort=relevance&size=100&ac=no) |
| 5 | address[pt] OR "autobiography"[pt] OR "bibliography"[pt] OR "biography"[pt] OR congress[pt] OR "dictionary"[pt] OR "directory"[pt] OR "festschrift"[pt] OR "historical article"[pt] OR lecture[pt] OR "legal case"[pt] OR "legislation"[pt] OR "periodical index"[pt] OR rats[tw] OR cow[tw] OR cows[tw] OR chicken[tw] OR chickens[tw] OR horse[tw] OR horses[tw] OR mice[tw] OR mouse[tw] OR bovine[tw] OR sheep[tw] OR ovine OR murine[tw] OR murinae[tw] OR "Self-management"[tw] OR "disease management"[tw] OR protocol[tw] | [5,192,842](https://pubmed.ncbi.nlm.nih.gov/?term=address%5Bpt%5D+OR+%22autobiography%22%5Bpt%5D+OR+%22bibliography%22%5Bpt%5D+OR+%22biography%22%5Bpt%5D+OR+congress%5Bpt%5D+OR+%22dictionary%22%5Bpt%5D+OR+%22directory%22%5Bpt%5D+OR+%22festschrift%22%5Bpt%5D+OR+%22historical+article%22%5Bpt%5D+OR+lecture%5Bpt%5D+OR+%22legal+case%22%5Bpt%5D+OR+%22legislation%22%5Bpt%5D+OR+%22periodical+index%22%5Bpt%5D+OR+rats%5Btw%5D+OR+cow%5Btw%5D+OR+cows%5Btw%5D+OR+chicken%5Btw%5D+OR+chickens%5Btw%5D+OR+horse%5Btw%5D+OR+horses%5Btw%5D+OR+mice%5Btw%5D+OR+mouse%5Btw%5D+OR+bovine%5Btw%5D+OR+sheep%5Btw%5D+OR+ovine+OR+murine%5Btw%5D+OR+murinae%5Btw%5D+OR+%22Self-management%22%5Btw%5D+OR+%22disease+management%22%5Btw%5D+OR+protocol%5Btw%5D&sort=relevance&size=100&ac=no) |
| 6 | #4 NOT #5 | [301,424](https://pubmed.ncbi.nlm.nih.gov/?term=%234+NOT+%235&sort=relevance&size=100&ac=no) |
| 7 | ("preventive service*"[tw] OR colonoscopy[tiab] OR lifestyle*[tiab] OR smoking[tiab] OR tobacco[tiab] OR obesity[tiab] OR cholesterol[tiab] OR alcohol*[tiab] OR aspirin[tiab] OR "blood pressure"[tiab] OR hypertension[tiab] OR "breast cancer"[tiab] OR "cervical cancer"[tiab] OR "colon cancer"[tiab] OR depression[tiab] OR diabetes[tiab] OR Falls[tiab] OR "substance abuse"[tiab] OR HIV[tiab] OR "intimate partner violence"[tiab] OR "domestic violence"[tiab] OR "healthy diet"[tiab] OR "physical activity"[tiab] OR exercise[tiab] OR "lung cancer"[tiab] OR osteoporosis[tiab]) | [3,996,862](https://pubmed.ncbi.nlm.nih.gov/?term=%28%22preventive+service%2A%22%5Btw%5D+OR+colonoscopy%5Btiab%5D+OR+lifestyle%2A%5Btiab%5D+OR+smoking%5Btiab%5D+OR+tobacco%5Btiab%5D+OR+obesity%5Btiab%5D+OR+cholesterol%5Btiab%5D+OR+alcohol%2A%5Btiab%5D+OR+aspirin%5Btiab%5D+OR+%22blood+pressure%22%5Btiab%5D+OR+hypertension%5Btiab%5D+OR+%22breast+cancer%22%5Btiab%5D+OR+%22cervical+cancer%22%5Btiab%5D+OR+%22colon+cancer%22%5Btiab%5D+OR+depression%5Btiab%5D+OR+diabetes%5Btiab%5D+OR+Falls%5Btiab%5D+OR+%22substance+abuse%22%5Btiab%5D+OR+HIV%5Btiab%5D+OR+%22intimate+partner+violence%22%5Btiab%5D+OR+%22domestic+violence%22%5Btiab%5D+OR+%22healthy+diet%22%5Btiab%5D+OR+%22physical+activity%22%5Btiab%5D+OR+exercise%5Btiab%5D+OR+%22lung+cancer%22%5Btiab%5D+OR+osteoporosis%5Btiab%5D%29&sort=relevance&size=100&ac=no) |
| 8 | #6 AND #7 | [86,112](https://pubmed.ncbi.nlm.nih.gov/?term=%236+AND+%237&sort=relevance&size=100&ac=no) |
| 9 | "Health Equity"[Mesh:noexp] OR "Health Status Disparities"[Mesh:noexp] OR "Minority health"[Mesh:noexp] OR Prejudice[Mesh:noexp] OR "Psychosocial Deprivation"[Mesh:noexp] OR "Racial Groups"[Mesh] OR Racism[Mesh:noexp] OR "Social determinants of Health"[Mesh:noexp] OR "Social Discrimination"[Mesh:noexp] OR Xenophobia[Mesh:noexp] OR disparit*[tiab] OR equity[tiab] OR ethnic*[tw] OR ethnology[tw] OR inequit*[tiab] OR "foreign language"[tw] OR "health*care disparit*"[tw] OR "healthcare disparit*"[tw] OR "health status disparit*"[tw] OR "health disparit*"[tw] OR "health inequalit*"[tw] OR "health inequit*"[tw] OR "health equit*"[tw] OR "health equalit*"[tw] | [536,364](https://pubmed.ncbi.nlm.nih.gov/?term=%22Health+Equity%22%5BMesh%3Anoexp%5D+OR+%22Health+Status+Disparities%22%5BMesh%3Anoexp%5D+OR+%22Minority+health%22%5BMesh%3Anoexp%5D+OR+Prejudice%5BMesh%3Anoexp%5D+OR+%22Psychosocial+Deprivation%22%5BMesh%3Anoexp%5D+OR+%22Racial+Groups%22%5BMesh%5D+OR+Racism%5BMesh%3Anoexp%5D+OR+%22Social+determinants+of+Health%22%5BMesh%3Anoexp%5D+OR+%22Social+Discrimination%22%5BMesh%3Anoexp%5D+OR+Xenophobia%5BMesh%3Anoexp%5D+OR+disparit%2A%5Btiab%5D+OR+equity%5Btiab%5D+OR+ethnic%2A%5Btw%5D+OR+ethnology%5Btw%5D+OR+inequit%2A%5Btiab%5D+OR+%22foreign+language%22%5Btw%5D+OR+%22health%2Acare+disparit%2A%22%5Btw%5D+OR+%22healthcare+disparit%2A%22%5Btw%5D+OR+%22health+status+disparit%2A%22%5Btw%5D+OR+%22health+disparit%2A%22%5Btw%5D+OR+%22health+inequalit%2A%22%5Btw%5D+OR+%22health+inequit%2A%22%5Btw%5D+OR+%22health+equit%2A%22%5Btw%5D+OR+%22health+equalit%2A%22%5Btw%5D&sort=relevance&size=100&ac=no) |
| 10 | #8 AND #9 | [8,404](https://pubmed.ncbi.nlm.nih.gov/?term=%238+AND+%239&sort=relevance&size=100&ac=no) |
| 11 | #10 AND (incidence OR morbidity OR mortality) | [5,563](https://pubmed.ncbi.nlm.nih.gov/?term=%2310+AND+%28incidence+OR+morbidity+OR+mortality%29&sort=relevance&size=100&ac=no) |
| 12 | #10 AND (incidence OR morbidity OR mortality) Filters: Review | [485](https://pubmed.ncbi.nlm.nih.gov/?term=%2310+AND+%28incidence+OR+morbidity+OR+mortality%29&filter=pubt.review&ac=no&size=100&sort=relevance) |
| 13 | #10 AND (incidence OR morbidity OR mortality) Filters: Meta-Analysis, Review, Systematic Review | [559](https://pubmed.ncbi.nlm.nih.gov/?term=%2310+AND+%28incidence+OR+morbidity+OR+mortality%29&filter=pubt.meta-analysis&filter=pubt.review&ac=no&size=100&sort=relevance) |
| 14 | #10 AND (incidence OR morbidity OR mortality) Filters: Case Reports | [8](https://pubmed.ncbi.nlm.nih.gov/?term=%2310+AND+%28incidence+OR+morbidity+OR+mortality%29&filter=pubt.casereports&ac=no&size=100&sort=relevance) |
| 15 | #10 AND (incidence OR morbidity OR mortality) Filters: Case Reports, Editorial | [59](https://pubmed.ncbi.nlm.nih.gov/?term=%2310+AND+%28incidence+OR+morbidity+OR+mortality%29&filter=pubt.casereports&filter=pubt.editorial&ac=no&size=100&sort=relevance) |
| 16 | #10 AND (incidence OR morbidity OR mortality) Filters: Case Reports, Editorial, Letter | [92](https://pubmed.ncbi.nlm.nih.gov/?term=%2310+AND+%28incidence+OR+morbidity+OR+mortality%29&filter=pubt.casereports&filter=pubt.editorial&filter=pubt.letter&ac=no&size=100&sort=relevance) |
| 17 | #10 AND (uptake[tiab] OR utilization[tiab] OR (access[tiab] AND screen*[tiab])) | [1,720](https://pubmed.ncbi.nlm.nih.gov/?term=%2310+AND+%28uptake%5Btiab%5D+OR+utilization%5Btiab%5D+OR+%28access%5Btiab%5D+AND+screen%2A%5Btiab%5D%29%29&sort=relevance&size=100&ac=no) |
| 18 | #10 AND (uptake[tiab] OR utilization[tiab] OR (access[tiab] AND screen*[tiab])) Filters: Review | [163](https://pubmed.ncbi.nlm.nih.gov/?term=%2310+AND+%28uptake%5Btiab%5D+OR+utilization%5Btiab%5D+OR+%28access%5Btiab%5D+AND+screen%2A%5Btiab%5D%29%29&filter=pubt.review&ac=no&size=100&sort=relevance) |
| 19 | #10 AND (uptake[tiab] OR utilization[tiab] OR (access[tiab] AND screen*[tiab])) Filters: Meta-Analysis, Review, Systematic Review | [193](https://pubmed.ncbi.nlm.nih.gov/?term=%2310+AND+%28uptake%5Btiab%5D+OR+utilization%5Btiab%5D+OR+%28access%5Btiab%5D+AND+screen%2A%5Btiab%5D%29%29&filter=pubt.meta-analysis&filter=pubt.review&ac=no&size=100&sort=relevance) |
| 20 | #10 AND (uptake[tiab] OR utilization[tiab] OR (access[tiab] AND screen*[tiab])) Filters: Case Reports | [1](https://pubmed.ncbi.nlm.nih.gov/?term=%2310+AND+%28uptake%5Btiab%5D+OR+utilization%5Btiab%5D+OR+%28access%5Btiab%5D+AND+screen%2A%5Btiab%5D%29%29&filter=pubt.casereports&ac=no&size=100&sort=relevance) |
| 21 | #10 AND (uptake[tiab] OR utilization[tiab] OR (access[tiab] AND screen*[tiab])) Filters: Editorial | [6](https://pubmed.ncbi.nlm.nih.gov/?term=%2310+AND+%28uptake%5Btiab%5D+OR+utilization%5Btiab%5D+OR+%28access%5Btiab%5D+AND+screen%2A%5Btiab%5D%29%29&filter=pubt.editorial&ac=no&size=100&sort=relevance) |
| 22 | #10 AND (uptake[tiab] OR utilization[tiab] OR (access[tiab] AND screen*[tiab])) Filters: Editorial, Letter | [8](https://pubmed.ncbi.nlm.nih.gov/?term=%2310+AND+%28uptake%5Btiab%5D+OR+utilization%5Btiab%5D+OR+%28access%5Btiab%5D+AND+screen%2A%5Btiab%5D%29%29&filter=pubt.editorial&filter=pubt.letter&ac=no&size=100&sort=relevance) |
| 23 | #10 AND Cause*[tw] | [592](https://pubmed.ncbi.nlm.nih.gov/?term=%2310+AND+Cause%2A%5Btw%5D&sort=relevance&size=100&ac=no) |
| 24 | #10 AND Cause*[tw] Filters: Review | [108](https://pubmed.ncbi.nlm.nih.gov/?term=%2310+AND+Cause%2A%5Btw%5D&filter=pubt.review&ac=no&size=100&sort=relevance) |
| 25 | #10 AND Cause*[tw] Filters: Meta-Analysis, Review, Systematic Review | [120](https://pubmed.ncbi.nlm.nih.gov/?term=%2310+AND+Cause%2A%5Btw%5D&filter=pubt.meta-analysis&filter=pubt.review&ac=no&size=100&sort=relevance) |
| 26 | #10 AND Cause*[tw] Filters: Case Reports | [1](https://pubmed.ncbi.nlm.nih.gov/?term=%2310+AND+Cause%2A%5Btw%5D&filter=pubt.casereports&ac=no&size=100&sort=relevance) |
| 27 | #10 AND Cause*[tw] Filters: Editorial | [10](https://pubmed.ncbi.nlm.nih.gov/?term=%2310+AND+Cause%2A%5Btw%5D&filter=pubt.editorial&ac=no&size=100&sort=relevance) |
| 28 | #10 AND Cause*[tw] Filters: Editorial, Letter | [11](https://pubmed.ncbi.nlm.nih.gov/?term=%2310+AND+Cause%2A%5Btw%5D&filter=pubt.editorial&filter=pubt.letter&ac=no&size=100&sort=relevance) |
| 29 | "Patient-Centered Care"[Mesh] OR (("patient-centered"[tiab] OR "patient-focused"[tiab] OR "person-centered"[tiab]) AND care[tiab]) OR "Precision Medicine"[Majr] OR ((individual*[tiab] OR Individualize*[tiab] OR holistic[tiab] OR "whole person"[tiab] OR personalized[tiab]) AND care[tiab]) OR "patient needs"[tiab] OR "patient values"[tiab] OR "Physician-Patient Relations"[Majr] OR "Doctor-patient relation*"[tiab] OR "Professional-Patient Relations"[Majr] OR "Patient Preference"[Majr] OR "patient preference*"[tiab] OR "social competenc*"[tiab] OR "Decision Making, Shared"[Majr] OR "shared decision making"[tiab] OR "Patient Self-Determination Act"[Majr] OR "patient decision making"[tiab] OR "patient engagement"[tiab] OR "patient involvement"[tiab] OR "patient empowerment"[tiab] OR "patient partnership"[tiab] OR "patient activation" [tiab] OR "patient-activated"[tiab] OR "Patient Acceptance of Health Care"[Majr] OR "consumer participation"[title] OR "consumer engagement"[tiab] OR "consumer involvement"[tiab] OR "consumer empowerment"[tiab] OR "consumer partnership"[tiab] OR "consumer activation"[tiab] OR "patient context"[tiab] OR "integrated care"[tiab] OR "coordinated care"[tiab] OR "Care coordination"[tiab] OR "continuity of care"[tiab] OR "healthcare teams"[tiab] OR "team-based care"[tiab] OR teamwork[tiab] | [473,922](https://pubmed.ncbi.nlm.nih.gov/?term=%22Patient-Centered+Care%22%5BMesh%5D+OR+%28%28%22patient-centered%22%5Btiab%5D+OR+%22patient-focused%22%5Btiab%5D+OR+%22person-centered%22%5Btiab%5D%29+AND+care%5Btiab%5D%29+OR+%22Precision+Medicine%22%5BMajr%5D+OR+%28%28individual%2A%5Btiab%5D+OR+Individualize%2A%5Btiab%5D+OR+holistic%5Btiab%5D+OR+%22whole+person%22%5Btiab%5D+OR+personalized%5Btiab%5D%29+AND+care%5Btiab%5D%29+OR+%22patient+needs%22%5Btiab%5D+OR+%22patient+values%22%5Btiab%5D+OR+%22Physician-Patient+Relations%22%5BMajr%5D+OR+%22Doctor-patient+relation%2A%22%5Btiab%5D+OR+%22Professional-Patient+Relations%22%5BMajr%5D+OR+%22Patient+Preference%22%5BMajr%5D+OR+%22patient+preference%2A%22%5Btiab%5D+OR+%22social+competenc%2A%22%5Btiab%5D+OR+%22Decision+Making%2C+Shared%22%5BMajr%5D+OR+%22shared+decision+making%22%5Btiab%5D+OR+%22Patient+Self-Determination+Act%22%5BMajr%5D+OR+%22patient+decision+making%22%5Btiab%5D+OR+%22patient+engagement%22%5Btiab%5D+OR+%22patient+involvement%22%5Btiab%5D+OR+%22patient+empowerment%22%5Btiab%5D+OR+%22patient+partnership%22%5Btiab%5D+OR+%22patient+activation%22+%5Btiab%5D+OR+%22patient-activated%22%5Btiab%5D+OR+%22Patient+Acceptance+of+Health+Care%22%5BMajr%5D+OR+%22consumer+participation%22%5Btitle%5D+OR+%22consumer+engagement%22%5Btiab%5D+OR+%22consumer+involvement%22%5Btiab%5D+OR+%22consumer+empowerment%22%5Btiab%5D+OR+%22consumer+partnership%22%5Btiab%5D+OR+%22consumer+activation%22%5Btiab%5D+OR+%22patient+context%22%5Btiab%5D+OR+%22integrated+care%22%5Btiab%5D+OR+%22coordinated+care%22%5Btiab%5D+OR+%22Care+coordination%22%5Btiab%5D+OR+%22continuity+of+care%22%5Btiab%5D+OR+%22healthcare+teams%22%5Btiab%5D+OR+%22team-based+care%22%5Btiab%5D+OR+teamwork%5Btiab%5D&sort=relevance&size=100&ac=no) |
| 30 | #10 AND #29 | [1,569](https://pubmed.ncbi.nlm.nih.gov/?term=%2310+AND+%2329&sort=relevance&size=100&ac=no) |
| 31 | #10 AND #29 Filters: Review | [133](https://pubmed.ncbi.nlm.nih.gov/?term=%2310+AND+%2329&filter=pubt.review&ac=no&size=100&sort=relevance) |
| 32 | #10 AND #29 Filters: Meta-Analysis, Review, Systematic Review | [158](https://pubmed.ncbi.nlm.nih.gov/?term=%2310+AND+%2329&filter=pubt.meta-analysis&filter=pubt.review&ac=no&size=100&sort=relevance) |
| 33 | #10 AND #29 Filters: Case Reports | [1](https://pubmed.ncbi.nlm.nih.gov/?term=%2310+AND+%2329&filter=pubt.casereports&ac=no&size=100&sort=relevance) |
| 34 | #10 AND #29 Filters: Editorial | [9](https://pubmed.ncbi.nlm.nih.gov/?term=%2310+AND+%2329&filter=pubt.editorial&ac=no&size=100&sort=relevance) |
| 35 | #10 AND #29 Filters: Editorial, Letter | [11](https://pubmed.ncbi.nlm.nih.gov/?term=%2310+AND+%2329&filter=pubt.editorial&filter=pubt.letter&ac=no&size=100&sort=relevance) |
| 36 | ("Organizational Innovation"[Majr] OR "Models, Organizational"[Majr] OR "care model*"[tiab] OR "service"[tiab] OR ("Delivery of Health Care"[Majr] AND model*[tw]) OR program[tiab] OR programmatic[tiab] OR redesign[tiab] OR transformation[tiab] OR innovation[tiab] OR innovative[tiab] OR "new model"[tiab] OR reform[title] OR "quality"[title] OR "healthcare improvement"[tiab] OR "system improvement"[tiab] OR strategy[title] OR strategies[title] OR "improve care"[title] OR "care improvement"[title] OR "care delivery"[title] OR "Health Systems Agencies"[Majr] OR "Social Determinants of Health"[Mesh] OR "Patient Care Bundles"[Mesh] OR "Patient Reported Outcome Measures"[Majr] OR "Quality Measure*"[tiab] OR "Healthy People Programs"[Majr]) | [1,751,457](https://pubmed.ncbi.nlm.nih.gov/?term=%28%22Organizational+Innovation%22%5BMajr%5D+OR+%22Models%2C+Organizational%22%5BMajr%5D+OR+%22care+model%2A%22%5Btiab%5D+OR+%22service%22%5Btiab%5D+OR+%28%22Delivery+of+Health+Care%22%5BMajr%5D+AND+model%2A%5Btw%5D%29+OR+program%5Btiab%5D+OR+programmatic%5Btiab%5D+OR+redesign%5Btiab%5D+OR+transformation%5Btiab%5D+OR+innovation%5Btiab%5D+OR+innovative%5Btiab%5D+OR+%22new+model%22%5Btiab%5D+OR+reform%5Btitle%5D+OR+%22quality%22%5Btitle%5D+OR+%22healthcare+improvement%22%5Btiab%5D+OR+%22system+improvement%22%5Btiab%5D+OR+strategy%5Btitle%5D+OR+strategies%5Btitle%5D+OR+%22improve+care%22%5Btitle%5D+OR+%22care+improvement%22%5Btitle%5D+OR+%22care+delivery%22%5Btitle%5D+OR+%22Health+Systems+Agencies%22%5BMajr%5D+OR+%22Social+Determinants+of+Health%22%5BMesh%5D+OR+%22Patient+Care+Bundles%22%5BMesh%5D+OR+%22Patient+Reported+Outcome+Measures%22%5BMajr%5D+OR+%22Quality+Measure%2A%22%5Btiab%5D+OR+%22Healthy+People+Programs%22%5BMajr%5D%29&sort=relevance&size=100&ac=no) |
| 37 | #10 AND #36 | [2,885](https://pubmed.ncbi.nlm.nih.gov/?term=%2310+AND+%2336&sort=relevance&size=100&ac=no) |
| 38 | #10 AND #36 Filters: Review | [213](https://pubmed.ncbi.nlm.nih.gov/?term=%2310+AND+%2336&filter=pubt.review&ac=no&size=100&sort=relevance) |
| 39 | #10 AND #36 Filters: Review, Systematic Review | [232](https://pubmed.ncbi.nlm.nih.gov/?term=%2310+AND+%2336&filter=pubt.review&filter=pubt.systematicreview&ac=no&size=100&sort=relevance) |
| 40 | #10 AND #36 Filters: Meta-Analysis, Review, Systematic Review | [235](https://pubmed.ncbi.nlm.nih.gov/?term=%2310+AND+%2336&filter=pubt.meta-analysis&filter=pubt.review&filter=pubt.systematicreview&ac=no&size=100&sort=relevance) |
| 41 | #10 AND #36 Filters: Case Reports | [4](https://pubmed.ncbi.nlm.nih.gov/?term=%2310+AND+%2336&filter=pubt.casereports&ac=no&size=100&sort=relevance) |
| 42 | Search#10 AND #36 Filters: Editorial | [13](https://pubmed.ncbi.nlm.nih.gov/?term=%2310+AND+%2336&filter=pubt.editorial&ac=no&size=100&sort=relevance) |
| 43 | #10 AND #36 Filters: Editorial, Letter | [17](https://pubmed.ncbi.nlm.nih.gov/?term=%2310+AND+%2336&filter=pubt.editorial&filter=pubt.letter&ac=no&size=100&sort=relevance) |
| 44 | #10 AND (policy[tw] AND intervention*[tw]) | [263](https://pubmed.ncbi.nlm.nih.gov/?term=%2310+AND+%28policy%5Btw%5D+AND+intervention%2A%5Btw%5D%29&sort=relevance&size=100&ac=no) |
| 45 | #10 AND (policy[tw] AND intervention*[tw]) Filters: Review | [51](https://pubmed.ncbi.nlm.nih.gov/?term=%2310+AND+%28policy%5Btw%5D+AND+intervention%2A%5Btw%5D%29&filter=pubt.review&ac=no&size=100&sort=relevance) |
| 46 | #10 AND (policy[tw] AND intervention*[tw]) Filters: Review, Systematic Review | [56](https://pubmed.ncbi.nlm.nih.gov/?term=%2310+AND+%28policy%5Btw%5D+AND+intervention%2A%5Btw%5D%29&filter=pubt.review&filter=pubt.systematicreview&ac=no&size=100&sort=relevance) |
| 47 | #10 AND (policy[tw] AND intervention*[tw]) Filters: Meta-Analysis, Review, Systematic Review | [56](https://pubmed.ncbi.nlm.nih.gov/?term=%2310+AND+%28policy%5Btw%5D+AND+intervention%2A%5Btw%5D%29&filter=pubt.meta-analysis&filter=pubt.review&filter=pubt.systematicreview&ac=no&size=100&sort=relevance) |
| 48 | #10 AND (policy[tw] AND intervention*[tw]) Filters: Case Reports | 0 |
| 49 | #10 AND (policy[tw] AND intervention*[tw]) Filters: Editorial | [9](https://pubmed.ncbi.nlm.nih.gov/?term=%2310+AND+%28policy%5Btw%5D+AND+intervention%2A%5Btw%5D%29&filter=pubt.editorial&ac=no&size=100&sort=relevance) |
| 50 | #10 AND (policy[tw] AND intervention*[tw]) Filters: Editorial, Letter | [10](https://pubmed.ncbi.nlm.nih.gov/?term=%2310+AND+%28policy%5Btw%5D+AND+intervention%2A%5Btw%5D%29&filter=pubt.editorial&filter=pubt.letter&ac=no&size=100&sort=relevance) |

| Search Number | PubMed Query (RCTs) | Results |
| --- | --- | --- |
| 1 | "Preventive Health Services"[Majr] OR "clinical preventive services"[tiab] OR "clinical preventive service"[tiab] OR Preventive[title] OR Prevention[title] OR Mass Screening[Majr] OR screening[title] OR screen*[title] OR screens[title] OR screened[title] OR Counseling[Majr] OR counsel*[title] OR counseling[title] OR Immunization[Majr] OR Vaccination[Majr] OR "Immunization Programs"[Majr] OR immunization[title] OR vaccine*[title] OR vaccination*[title] OR "Primary Prevention"[Majr] | [929,247](https://pubmed.ncbi.nlm.nih.gov/?term=%22Preventive+Health+Services%22%5BMajr%5D+OR+%22clinical+preventive+services%22%5Btiab%5D+OR+%22clinical+preventive+service%22%5Btiab%5D+OR+Preventive%5Btitle%5D+OR+Prevention%5Btitle%5D+OR+Mass+Screening%5BMajr%5D+OR+screening%5Btitle%5D+OR+screen%2A%5Btitle%5D+OR+screens%5Btitle%5D+OR+screened%5Btitle%5D+OR+Counseling%5BMajr%5D+OR+counsel%2A%5Btitle%5D+OR+counseling%5Btitle%5D+OR+Immunization%5BMajr%5D+OR+Vaccination%5BMajr%5D+OR+%22Immunization+Programs%22%5BMajr%5D+OR+immunization%5Btitle%5D+OR+vaccine%2A%5Btitle%5D+OR+vaccination%2A%5Btitle%5D+OR+%22Primary+Prevention%22%5BMajr%5D&sort=relevance&size=100&ac=no) |
| 2 | "Preventive Health Services"[Majr] OR "clinical preventive services"[tiab] OR "clinical preventive service"[tiab] OR Preventive[title] OR Prevention[title] OR Mass Screening[Majr] OR screening[title] OR screen*[title] OR screens[title] OR screened[title] OR Counseling[Majr] OR counsel*[title] OR counseling[title] OR Immunization[Majr] OR Vaccination[Majr] OR "Immunization Programs"[Majr] OR immunization[title] OR vaccine*[title] OR vaccination*[title] OR "Primary Prevention"[Majr] Filters: English | [801,136](https://pubmed.ncbi.nlm.nih.gov/?term=%22Preventive+Health+Services%22%5BMajr%5D+OR+%22clinical+preventive+services%22%5Btiab%5D+OR+%22clinical+preventive+service%22%5Btiab%5D+OR+Preventive%5Btitle%5D+OR+Prevention%5Btitle%5D+OR+Mass+Screening%5BMajr%5D+OR+screening%5Btitle%5D+OR+screen%2A%5Btitle%5D+OR+screens%5Btitle%5D+OR+screened%5Btitle%5D+OR+Counseling%5BMajr%5D+OR+counsel%2A%5Btitle%5D+OR+counseling%5Btitle%5D+OR+Immunization%5BMajr%5D+OR+Vaccination%5BMajr%5D+OR+%22Immunization+Programs%22%5BMajr%5D+OR+immunization%5Btitle%5D+OR+vaccine%2A%5Btitle%5D+OR+vaccination%2A%5Btitle%5D+OR+%22Primary+Prevention%22%5BMajr%5D&filter=lang.english&ac=no&size=100&sort=relevance) |
| 3 | "Preventive Health Services"[Majr] OR "clinical preventive services"[tiab] OR "clinical preventive service"[tiab] OR Preventive[title] OR Prevention[title] OR Mass Screening[Majr] OR screening[title] OR screen*[title] OR screens[title] OR screened[title] OR Counseling[Majr] OR counsel*[title] OR counseling[title] OR Immunization[Majr] OR Vaccination[Majr] OR "Immunization Programs"[Majr] OR immunization[title] OR vaccine*[title] OR vaccination*[title] OR "Primary Prevention"[Majr] Filters: English, from 2012 - 2023 | [388,862](https://pubmed.ncbi.nlm.nih.gov/?term=%22Preventive+Health+Services%22%5BMajr%5D+OR+%22clinical+preventive+services%22%5Btiab%5D+OR+%22clinical+preventive+service%22%5Btiab%5D+OR+Preventive%5Btitle%5D+OR+Prevention%5Btitle%5D+OR+Mass+Screening%5BMajr%5D+OR+screening%5Btitle%5D+OR+screen%2A%5Btitle%5D+OR+screens%5Btitle%5D+OR+screened%5Btitle%5D+OR+Counseling%5BMajr%5D+OR+counsel%2A%5Btitle%5D+OR+counseling%5Btitle%5D+OR+Immunization%5BMajr%5D+OR+Vaccination%5BMajr%5D+OR+%22Immunization+Programs%22%5BMajr%5D+OR+immunization%5Btitle%5D+OR+vaccine%2A%5Btitle%5D+OR+vaccination%2A%5Btitle%5D+OR+%22Primary+Prevention%22%5BMajr%5D&filter=lang.english&filter=years.2012-2023&ac=no&size=100&sort=relevance) |
| 4 | #3 NOT (("Adolescent"[Mesh] OR "Child"[Mesh] OR "Infant"[Mesh]) NOT "Adult"[Mesh]) | [346,684](https://pubmed.ncbi.nlm.nih.gov/?term=%233+NOT+%28%28%22Adolescent%22%5BMesh%5D+OR+%22Child%22%5BMesh%5D+OR+%22Infant%22%5BMesh%5D%29+NOT+%22Adult%22%5BMesh%5D%29&sort=relevance&size=100&ac=no) |
| 5 | address[pt] OR "autobiography"[pt] OR "bibliography"[pt] OR "biography"[pt] OR congress[pt] OR "dictionary"[pt] OR "directory"[pt] OR "festschrift"[pt] OR "historical article"[pt] OR lecture[pt] OR "legal case"[pt] OR "legislation"[pt] OR "periodical index"[pt] OR rats[tw] OR cow[tw] OR cows[tw] OR chicken[tw] OR chickens[tw] OR horse[tw] OR horses[tw] OR mice[tw] OR mouse[tw] OR bovine[tw] OR sheep[tw] OR ovine OR murine[tw] OR murinae[tw] OR "Self-management"[tw] OR "disease management"[tw] OR protocol[tw] | [5,192,897](https://pubmed.ncbi.nlm.nih.gov/?term=address%5Bpt%5D+OR+%22autobiography%22%5Bpt%5D+OR+%22bibliography%22%5Bpt%5D+OR+%22biography%22%5Bpt%5D+OR+congress%5Bpt%5D+OR+%22dictionary%22%5Bpt%5D+OR+%22directory%22%5Bpt%5D+OR+%22festschrift%22%5Bpt%5D+OR+%22historical+article%22%5Bpt%5D+OR+lecture%5Bpt%5D+OR+%22legal+case%22%5Bpt%5D+OR+%22legislation%22%5Bpt%5D+OR+%22periodical+index%22%5Bpt%5D+OR+rats%5Btw%5D+OR+cow%5Btw%5D+OR+cows%5Btw%5D+OR+chicken%5Btw%5D+OR+chickens%5Btw%5D+OR+horse%5Btw%5D+OR+horses%5Btw%5D+OR+mice%5Btw%5D+OR+mouse%5Btw%5D+OR+bovine%5Btw%5D+OR+sheep%5Btw%5D+OR+ovine+OR+murine%5Btw%5D+OR+murinae%5Btw%5D+OR+%22Self-management%22%5Btw%5D+OR+%22disease+management%22%5Btw%5D+OR+protocol%5Btw%5D&sort=relevance&size=100&ac=no) |
| 6 | #4 NOT #5 | [301,462](https://pubmed.ncbi.nlm.nih.gov/?term=%234+NOT+%235&sort=relevance&size=100&ac=no) |
| 7 | ("preventive service*"[tw] OR colonoscopy[tiab] OR lifestyle*[tiab] OR smoking[tiab] OR tobacco[tiab] OR obesity[tiab] OR cholesterol[tiab] OR alcohol*[tiab] OR aspirin[tiab] OR "blood pressure"[tiab] OR hypertension[tiab] OR "breast cancer"[tiab] OR "cervical cancer"[tiab] OR "colon cancer"[tiab] OR depression[tiab] OR diabetes[tiab] OR Falls[tiab] OR "substance abuse"[tiab] OR HIV[tiab] OR "intimate partner violence"[tiab] OR "domestic violence"[tiab] OR "healthy diet"[tiab] OR "physical activity"[tiab] OR exercise[tiab] OR "lung cancer"[tiab] OR osteoporosis[tiab]) | [3,996,980](https://pubmed.ncbi.nlm.nih.gov/?term=%28%22preventive+service%2A%22%5Btw%5D+OR+colonoscopy%5Btiab%5D+OR+lifestyle%2A%5Btiab%5D+OR+smoking%5Btiab%5D+OR+tobacco%5Btiab%5D+OR+obesity%5Btiab%5D+OR+cholesterol%5Btiab%5D+OR+alcohol%2A%5Btiab%5D+OR+aspirin%5Btiab%5D+OR+%22blood+pressure%22%5Btiab%5D+OR+hypertension%5Btiab%5D+OR+%22breast+cancer%22%5Btiab%5D+OR+%22cervical+cancer%22%5Btiab%5D+OR+%22colon+cancer%22%5Btiab%5D+OR+depression%5Btiab%5D+OR+diabetes%5Btiab%5D+OR+Falls%5Btiab%5D+OR+%22substance+abuse%22%5Btiab%5D+OR+HIV%5Btiab%5D+OR+%22intimate+partner+violence%22%5Btiab%5D+OR+%22domestic+violence%22%5Btiab%5D+OR+%22healthy+diet%22%5Btiab%5D+OR+%22physical+activity%22%5Btiab%5D+OR+exercise%5Btiab%5D+OR+%22lung+cancer%22%5Btiab%5D+OR+osteoporosis%5Btiab%5D%29&sort=relevance&size=100&ac=no) |
| 8 | #6 AND #7 | [86,121](https://pubmed.ncbi.nlm.nih.gov/?term=%236+AND+%237&sort=relevance&size=100&ac=no) |
| 9 | "Health Equity"[Mesh:noexp] OR "Health Status Disparities"[Mesh:noexp] OR "Minority health"[Mesh:noexp] OR Prejudice[Mesh:noexp] OR "Psychosocial Deprivation"[Mesh:noexp] OR "Racial Groups"[Mesh] OR Racism[Mesh:noexp] OR "Social determinants of Health"[Mesh:noexp] OR "Social Discrimination"[Mesh:noexp] OR Xenophobia[Mesh:noexp] OR disparit*[tiab] OR equity[tiab] OR ethnic*[tw] OR ethnology[tw] OR inequit*[tiab] OR "foreign language"[tw] OR "health*care disparit*"[tw] OR "healthcare disparit*"[tw] OR "health status disparit*"[tw] OR "health disparit*"[tw] OR "health inequalit*"[tw] OR "health inequit*"[tw] OR "health equit*"[tw] OR "health equalit*"[tw] | [536,383](https://pubmed.ncbi.nlm.nih.gov/?term=%22Health+Equity%22%5BMesh%3Anoexp%5D+OR+%22Health+Status+Disparities%22%5BMesh%3Anoexp%5D+OR+%22Minority+health%22%5BMesh%3Anoexp%5D+OR+Prejudice%5BMesh%3Anoexp%5D+OR+%22Psychosocial+Deprivation%22%5BMesh%3Anoexp%5D+OR+%22Racial+Groups%22%5BMesh%5D+OR+Racism%5BMesh%3Anoexp%5D+OR+%22Social+determinants+of+Health%22%5BMesh%3Anoexp%5D+OR+%22Social+Discrimination%22%5BMesh%3Anoexp%5D+OR+Xenophobia%5BMesh%3Anoexp%5D+OR+disparit%2A%5Btiab%5D+OR+equity%5Btiab%5D+OR+ethnic%2A%5Btw%5D+OR+ethnology%5Btw%5D+OR+inequit%2A%5Btiab%5D+OR+%22foreign+language%22%5Btw%5D+OR+%22health%2Acare+disparit%2A%22%5Btw%5D+OR+%22healthcare+disparit%2A%22%5Btw%5D+OR+%22health+status+disparit%2A%22%5Btw%5D+OR+%22health+disparit%2A%22%5Btw%5D+OR+%22health+inequalit%2A%22%5Btw%5D+OR+%22health+inequit%2A%22%5Btw%5D+OR+%22health+equit%2A%22%5Btw%5D+OR+%22health+equalit%2A%22%5Btw%5D&sort=relevance&size=100&ac=no) |
| 10 | #8 AND #9 | [8,405](https://pubmed.ncbi.nlm.nih.gov/?term=%238+AND+%239&sort=relevance&size=100&ac=no) |
| 11 | #8 AND #9 Filters: from 2019/1/1 - 2023/12/31 | [3,285](https://pubmed.ncbi.nlm.nih.gov/?term=%238+AND+%239&filter=dates.2019%2F1%2F1-2023%2F12%2F31&ac=no&size=100&sort=relevance) |
| 12 | #8 AND #9 Filters: Randomized Controlled Trial, from 2019/1/1 - 2023/12/31 | [194](https://pubmed.ncbi.nlm.nih.gov/?term=%238+AND+%239&filter=pubt.randomizedcontrolledtrial&filter=dates.2019%2F1%2F1-2023%2F12%2F31&ac=no&size=100&sort=relevance) |

| Search Number | PsycInfo Query | Limiters/Expanders | Results |
| --- | --- | --- | --- |
| 1 | MM "Preventive Health Services" OR TI "clinical preventive services" OR AB "clinical preventive services" OR TI "clinical preventive service" OR AB "clinical preventive service" OR TI Preventive OR TI Prevention OR MM Screening OR TI screening OR TI screen* OR TI screens OR TI screened OR MM Counseling OR TI counsel* OR TI counseling OR MM Immunization OR MM Vaccination OR MM “Vaccination Attitudes” OR TI immunization OR TI vaccine* OR TI vaccination* OR TI "Primary Prevention" OR AB "Primary Prevention" | Expanders - Apply equivalent subjects Search modes - Find all my search terms | 92,571 |
| 2 | S1 | Limiters - Publication Year: 2012-2023; English; Language: English; Age Groups: Adulthood (18 yrs & older); Population Group: Human Expanders - Apply equivalent subjects Search modes - Find all my search terms | 20,412 |
| 3 | TX (rats OR cow OR cows OR chicken OR chickens OR horse OR horses OR mice OR mouse.mp OR bovine OR sheep OR ovine OR murine OR murinae OR Self-management OR "disease management" OR protocol) | Expanders - Apply equivalent subjects Search modes - Find all my search terms | 358,829 |
| 4 | S2 NOT S3 | Expanders - Apply equivalent subjects Search modes - Find all my search terms | 19,694 |
| 5 | S4 | Limiters - Document Type: Abstract Collection, Bibliography, Chapter, Clarification, Dissertation, Encyclopedia Entry, Erratum/Correction, Interview, Obituary, Poetry, Publication Information, Review-Book, Review-Media, Review-Software & Other Expanders - Apply equivalent subjects Search modes - Find all my search terms | 3,649 |
| 6 | S4 NOT S5 | Expanders - Apply equivalent subjects Search modes - Find all my search terms | 16,045 |
| 7 | TX preventive service* OR TX preventive health service* OR TI colonoscopy OR AB colonoscopy OR TI lifestyle* OR AB lifestyle* OR TI smoking OR AB smoking OR TI tobacco OR AB tobacco OR TI obesity OR AB obesity OR TI cholesterol OR AB cholesterol OR TI alcohol* OR AB alcohol* OR TI aspirin OR AB aspirin OR TI "blood pressure" OR AB “blood pressure” OR TI hypertension OR AB hypertension OR TI "breast cancer" OR AB “breast cancer” OR TI "cervical cancer" OR AB “cervical cancer” OR TI "colon cancer" OR AB “colon cancer” OR TI “colorectal cancer” OR AB “colorectal cancer” OR TI depression OR AB depression OR TI diabetes OR AB diabetes OR TI Falls OR AB Falls OR TI "substance abuse" OR AB “substance abuse” OR TI HIV OR AB HIV OR TI "intimate partner violence" OR AB “intimate partner violence” OR TI "domestic violence" OR AB “domestic violence” OR TI "healthy diet" OR AB “healthy diet” OR TI "physical activity” OR AB “physical activity” OR TI exercise OR AB exercise OR TI "lung cancer" OR AB “lung cancer” OR TI osteoporosis OR AB osteoporosis | Expanders - Apply equivalent subjects Search modes - Find all my search terms | 768,104 |
| 8 | S6 AND S7 | Expanders - Apply equivalent subjects Search modes - Find all my search terms | 6,323 |
| 9 | DE Equity OR DE "Health Disparities" OR DE "Mental Health Disparities" OR DE "Minority Stress" OR TX "Minority health" OR DE "Prejudice" OR TX "Psychosocial Deprivation" OR DE "Racial Identity" OR DE "Racism" OR DE "Internalized Racism" OR DE "Systemic Racism" OR TX "Social determinants of Health" OR DE "Socioeconomic Factors" OR DE "Economic Disadvantage" OR DE "Economic Resources" OR DE "Employment Status" OR DE "Income Level" OR DE "Social Class" OR DE "Social Disadvantage" OR DE "Socioeconomic Status" OR DE "Family Socioeconomic Status" OR DE "Income Level" OR DE "Social Class" OR DE "Social Discrimination" OR DE "Age Discrimination" OR DE "Disability Discrimination" OR DE "Employment Discrimination" OR DE "Intersectionality" OR DE "Race and Ethnic Discrimination" OR DE "Sex Discrimination" OR DE "Social Class Bias" OR DE "Stranger Reactions" OR TX Xenophobia OR TI disparit* OR AB disparit* OR TI equity OR AB equit* OR TX ethnic* OR TX ethnology OR TI inequit* OR AB inequit* OR TX "foreign language" OR TX "health*care disparit*" OR TX "healthcare disparit*" OR TX "health status disparit*" OR TX "health disparit*" OR TX "health inequalit*" OR TX "health inequit*" OR TX "health equit*" OR TX "health equalit*" | Expanders - Apply equivalent subjects Search modes - Find all my search terms | 332,020 |
| 10 | S8 AND S9 | Expanders - Apply equivalent subjects Search modes - Find all my search terms | 1,170 |
| 11 | TX (Incidence OR morbidity OR mortality) | Expanders - Apply equivalent subjects Search modes - Find all my search terms | 129,758 |
| 12 | S10 AND S11 | Expanders - Apply equivalent subjects Search modes - Find all my search terms | 179 |
| 13 |  | Limiters - Methodology: LITERATURE REVIEW, -Systematic Review, META ANALYSIS, METASYNTHESIS Expanders - Apply equivalent subjects Search modes - Find all my search terms | 205,967 |
| 14 |  | Limiters - Methodology: CLINICAL CASE STUDY, NONCLINICAL CASE STUDY Expanders - Apply equivalent subjects Search modes - Find all my search terms | 125,138 |
| 15 |  | Limiters - Document Type: Editorial, Letter Expanders - Apply equivalent subjects Search modes - Find all my search terms | 70,457 |
| 16 | S12 AND S13 | Expanders - Apply equivalent subjects Search modes - Find all my search terms | 5 |
| 17 | S12 AND S14 | Expanders - Apply equivalent subjects Search modes - Find all my search terms | 0 |
| 18 | S12 AND S15 | Expanders - Apply equivalent subjects Search modes - Find all my search terms | 0 |
| 19 | TI uptake OR AB uptake OR TI utilization OR AB utilization OR TI (access AND screen*) OR AB (access AND screen*) | Expanders - Apply equivalent subjects Search modes - Find all my search terms | 64,949 |
| 20 | S10 AND S19 | Expanders - Apply equivalent subjects Search modes - Find all my search terms | 328 |
| 21 | S13 AND S20 | Expanders - Apply equivalent subjects Search modes - Find all my search terms | 3 |
| 22 | S14 AND S20 | Expanders - Apply equivalent subjects Search modes - Find all my search terms | 3 |
| 23 | S15 AND S20 | Expanders - Apply equivalent subjects Search modes - Find all my search terms | 0 |
| 24 | DE "Causality" OR TX cause* | Expanders - Apply equivalent subjects Search modes - Find all my search terms | 252,235 |
| 25 | S10 AND S24 | Expanders - Apply equivalent subjects Search modes - Find all my search terms | 101 |
| 26 | S13 AND S25 | Expanders - Apply equivalent subjects Search modes - Find all my search terms | 1 |
| 27 | S14 AND S25 | Expanders - Apply equivalent subjects Search modes - Find all my search terms | 0 |
| 28 | S15 AND S25 | Expanders - Apply equivalent subjects Search modes - Find all my search terms | 0 |
| 29 | TX "Patient-Centered Care" OR TI ((patient-centered OR patient-focused OR person-centered) AND care) OR AB ((patient-centered OR patient-focused OR person-centered) AND care) OR DE "Precision Medicine" OR TI ((individual* OR Individualize* OR holistic OR "whole person" OR personalized) AND care) OR AB ((individual* OR Individualize* OR holistic OR "whole person" OR personalized) AND care) OR TI "patient needs" OR AB “patient needs” OR TI "patient values" OR AB “patient values” OR TX "Physician-Patient Relations" OR TI "Doctor-patient relation*" OR AB “Doctor-patient relation*” OR TX "Professional-Patient Relations" OR TX "Patient Preference" OR DE "Social Skills" OR TX "social competenc*" OR exp *"Decision Making, Shared"/ OR (DE "Decision Making" AND TX shared) OR TX "shared decision making" OR TX "Patient Self-Determination Act" OR TX "patient decision making" OR TX "patient engagement" OR TX "patient involvement" OR TX "patient empowerment" OR TX "patient partnership" OR TX "patient activation" OR TX “patient-activated” OR TX "Patient Acceptance of Health Care" OR TX "consumer participation" OR TX "consumer engagement" OR TX "consumer involvement" OR TX "consumer empowerment" OR TX "consumer partnership" OR TX "consumer activation" OR TX "patient context" OR TX "integrated care" OR TX "coordinated care" OR TX "Care coordination" OR TX "continuity of care" OR TX "healthcare teams" OR TX "team-based care" OR TX teamwork | Expanders - Apply equivalent subjects Search modes - Find all my search terms | 159,225 |
| 30 | S10 AND S29 | Expanders - Apply equivalent subjects Search modes - Find all my search terms | 281 |
| 31 | S13 AND S30 | Expanders - Apply equivalent subjects Search modes - Find all my search terms | 1 |
| 32 | S14 AND S30 | Expanders - Apply equivalent subjects Search modes - Find all my search terms | 1 |
| 33 | S15 AND S30 | Expanders - Apply equivalent subjects Search modes - Find all my search terms | 0 |
| 34 | DE "Organizational Change" OR TX "Organizational Innovation" OR DE "Organizational Characteristics" OR TX “organizational model” OR TX "care model*" OR service.ti,ab. OR (exp DE "Health Care Delivery" AND model*) OR TX program OR TX programmatic OR TX redesign OR TX transformation OR TX innovation OR TX innovative OR TX "new model" OR TI reform OR TI quality OR TX "healthcare improvement" OR TX "system improvement" OR TI strategy OR TI strategies OR TI "improve care" OR TI "care improvement" OR TI "care delivery" OR TX "Health Systems Agencies" OR TX "Social Determinants of Health" OR TX "Patient Care Bundles" OR DE "Patient Reported Outcome Measures" OR TX "Quality Measure*" OR TX "Healthy People Programs" | Expanders - Apply equivalent subjects Search modes - Find all my search terms | 876,809 |
| 35 | S10 AND S34 | Expanders - Apply equivalent subjects Search modes - Find all my search terms | 469 |
| 36 | S13 AND S35 | Expanders - Apply equivalent subjects Search modes - Find all my search terms | 4 |
| 37 | S14 AND S35 | Expanders - Apply equivalent subjects Search modes - Find all my search terms | 3 |
| 38 | S15 AND S35 | Expanders - Apply equivalent subjects Search modes - Find all my search terms | 0 |
| 39 | TX policy AND intervention* | Expanders - Apply equivalent subjects Search modes - Find all my search terms | 44,800 |
| 40 | TX S10 AND S39 | Expanders - Apply equivalent subjects Search modes - Find all my search terms | 63 |
| 41 | TX S13 AND S40 | Expanders - Apply equivalent subjects Search modes - Find all my search terms | 0 |
| 42 | TX S14 AND S40 | Expanders - Apply equivalent subjects Search modes - Find all my search terms | 0 |
| 43 | TX S15 AND S40 | Expanders - Apply equivalent subjects Search modes - Find all my search terms | 0 |

| Search | PsycInfo RCT Query | Limiters/Expanders | Results |
| --- | --- | --- | --- |
| 1 | MM "Preventive Health Services" OR TI "clinical preventive services" OR AB "clinical preventive services" OR TI "clinical preventive service" OR AB "clinical preventive service" OR TI Preventive OR TI Prevention OR MM Screening OR TI screening OR TI screen* OR TI screens OR TI screened OR MM Counseling OR TI counsel* OR TI counseling OR MM Immunization OR MM Vaccination OR MM “Vaccination Attitudes” OR TI immunization OR TI vaccine* OR TI vaccination* OR TI "Primary Prevention" OR AB "Primary Prevention" | Expanders - Apply equivalent subjects Search modes - Find all my search terms | 92,571 |
| 2 | S1 | Limiters - Publication Year: 2012-2023; English; Language: English; Age Groups: Adulthood (18 yrs & older); Population Group: Human Expanders - Apply equivalent subjects Search modes - Find all my search terms | 20,412 |
| 3 | TX (rats OR cow OR cows OR chicken OR chickens OR horse OR horses OR mice OR mouse.mp OR bovine OR sheep OR ovine OR murine OR murinae OR Self-management OR "disease management" OR protocol) | Expanders - Apply equivalent subjects Search modes - Find all my search terms | 358,829 |
| 4 | S2 NOT S3 | Expanders - Apply equivalent subjects Search modes - Find all my search terms | 19,694 |
| 5 | S4 | Limiters - Document Type: Abstract Collection, Bibliography, Chapter, Clarification, Dissertation, Encyclopedia Entry, Erratum/Correction, Interview, Obituary, Poetry, Publication Information, Review-Book, Review-Media, Review-Software & Other Expanders - Apply equivalent subjects Search modes - Find all my search terms | 3,649 |
| 6 | S4 NOT S5 | Expanders - Apply equivalent subjects Search modes - Find all my search terms | 16,045 |
| 7 | TX preventive service* OR TX preventive health service* OR TI colonoscopy OR AB colonoscopy OR TI lifestyle* OR AB lifestyle* OR TI smoking OR AB smoking OR TI tobacco OR AB tobacco OR TI obesity OR AB obesity OR TI cholesterol OR AB cholesterol OR TI alcohol* OR AB alcohol* OR TI aspirin OR AB aspirin OR TI "blood pressure" OR AB “blood pressure” OR TI hypertension OR AB hypertension OR TI "breast cancer" OR AB “breast cancer” OR TI "cervical cancer" OR AB “cervical cancer” OR TI "colon cancer" OR AB “colon cancer” OR TI “colorectal cancer” OR AB “colorectal cancer” OR TI depression OR AB depression OR TI diabetes OR AB diabetes OR TI Falls OR AB Falls OR TI "substance abuse" OR AB “substance abuse” OR TI HIV OR AB HIV OR TI "intimate partner violence" OR AB “intimate partner violence” OR TI "domestic violence" OR AB “domestic violence” OR TI "healthy diet" OR AB “healthy diet” OR TI "physical activity” OR AB “physical activity” OR TI exercise OR AB exercise OR TI "lung cancer" OR AB “lung cancer” OR TI osteoporosis OR AB osteoporosis | Expanders - Apply equivalent subjects Search modes - Find all my search terms | 768,104 |
| 8 | S6 AND S7 | Expanders - Apply equivalent subjects Search modes - Find all my search terms | 6,323 |
| 9 | DE Equity OR DE "Health Disparities" OR DE "Mental Health Disparities" OR DE "Minority Stress" OR TX "Minority health" OR DE "Prejudice" OR TX "Psychosocial Deprivation" OR DE "Racial Identity" OR DE "Racism" OR DE "Internalized Racism" OR DE "Systemic Racism" OR TX "Social determinants of Health" OR DE "Socioeconomic Factors" OR DE "Economic Disadvantage" OR DE "Economic Resources" OR DE "Employment Status" OR DE "Income Level" OR DE "Social Class" OR DE "Social Disadvantage" OR DE "Socioeconomic Status" OR DE "Family Socioeconomic Status" OR DE "Income Level" OR DE "Social Class" OR DE "Social Discrimination" OR DE "Age Discrimination" OR DE "Disability Discrimination" OR DE "Employment Discrimination" OR DE "Intersectionality" OR DE "Race and Ethnic Discrimination" OR DE "Sex Discrimination" OR DE "Social Class Bias" OR DE "Stranger Reactions" OR TX Xenophobia OR TI disparit* OR AB disparit* OR TI equity OR AB equit* OR TX ethnic* OR TX ethnology OR TI inequit* OR AB inequit* OR TX "foreign language" OR TX "health*care disparit*" OR TX "healthcare disparit*" OR TX "health status disparit*" OR TX "health disparit*" OR TX "health inequalit*" OR TX "health inequit*" OR TX "health equit*" OR TX "health equalit*" | Expanders - Apply equivalent subjects Search modes - Find all my search terms | 332,020 |
| 10 | S8 AND S9 | Expanders - Apply equivalent subjects Search modes - Find all my search terms | 1,170 |
| 11 | S10 | Limiters - Methodology: CLINICAL TRIAL Expanders - Apply equivalent subjects Search modes - Find all my search terms | 20 |
| 12 | S10 AND ((random* AND control*) OR single-blind OR double-blind OR triple-blind OR treble OR “random allocation”) | Expanders - Apply equivalent subjects Search modes - Find all my search terms | 25 |
| 13 | S11 OR S12 | Expanders - Apply equivalent subjects Search modes - Find all my search terms | 36 |

Disparities Grey Literature Sources Searched

| Source | Website Link |
| --- | --- |
| Advisory Committee on Immunization Practices (ACIP) | <https://www.cdc.gov/vaccines/acip/index.html> |
| Agency for Healthcare Research and Quality (AHRQ) | <https://www.ahrq.gov/> |
| American Cancer Society (ACS) | [www.cancer.org](http://www.cancer.org) |
| American College of Cardiology (ACC) | [www.acc.org](http://www.acc.org) |
| American College of Physicians (ACP) | [www.acponline.org](http://www.acponline.org) |
| American Heart Association (AHA) | [www.heart.org](http://www.heart.org) |
| Centers for Disease Control and Prevention (CDC) | <https://www.cdc.gov/> |
| Centers for Medicare & Medicaid Services (CMS) | [www.cms.gov](http://www.cms.gov) |
| Commonwealth Fund | [www.commonwealthfund.org](http://www.commonwealthfund.org) |
| Evaluation Officer Counsel | [www.evaluation.gov](http://www.evaluation.gov) |
| Institute for Healthcare Improvement (IHI) | [www.ihi.org](http://www.ihi.org) |
| Kaiser Family Foundation (KFF) | [www.kff.org](http://www.kff.org) |
| National Academy of Medicine (NAM) | <https://nam.edu/> |
| National Cancer Institute (NCI) | [www.cancer.gov](http://www.cancer.gov) |
| National Colorectal Cancer Roundtable | <https://nccrt.org/> |
| National HPV Vaccination Roundtable | <https://hpvroundtable.org/> |
| National Institutes of Health Office of Disease Prevention (NIH ODP) | <https://prevention.nih.gov> |
| National Institutes on Minority Health and Health Disparities (NIMHD) | [www.nimhd.nih.gov](http://www.nimhd.nih.gov) |
| National Lung Cancer Roundtable | <https://nlcrt.org> |
| National Navigation Roundtable | <https://navigationroundtable.org/> |
| Patient-Centered Outcomes Research Institute (PCORI) | [www.pcori.org](http://www.pcori.org) |
| The Guide to Community Preventive Services (CPSTF) | <https://www.thecommunityguide.org/> |
| The Pathways to Prevention (P2P) Program | <https://prevention.nih.gov/research-priorities/research-needs-and-gaps/pathways-prevention> |
| U.S. Preventive Service Task Force (USPSTF) | <https://www.uspreventiveservicestaskforce.org/uspstf/> |

Disparities Technical Expert Panel and Key Informants

The list of people who participated in the technical expert panel (TEP) or as key informants for the health disparities focus area is provided in Table A-10.

Table A-10. Health Disparities in Clinical Preventive Services Technical Expert Panel Members and Key Informants

| Name | Organization | Role | Type of Organization |
| --- | --- | --- | --- |
| Monica L. Baskin, PhD | University of Pittsburgh | Professor of Medicine; Associate Director for Community Outreach and Engagement and Health Equity Comprehensive Cancer Center | Research/academia |
| Mary Butler, PhD, MBA | University of Minnesota | Associate Professor, Division of Health Policy & Management; Co-Director Minnesota Evidence-based Practice Center | Research/academia |
| Loretta Christensen, MD, MBA, MSJ, FACS | Indian Health Service | Chief Medical Officer | Federal agency |
| Michael Currie, MPH, MBA | UnitedHealth Group | Senior Vice President and Chief Health Equity Officer | Payer |
| Esa Davis, MD, MPH, FAAFP | University of Maryland School of Medicine | Senior Associate Dean for Population Health and Community Medicine | USPSTF (current/ former member) |
| David Grossman, MD | Kaiser Foundation Health Plan | Senior Vice President of Social and Community Health | Healthcare system |
| Anjali Jain, MD | Evidence-Based Practice Center program, Agency for Healthcare Research and Quality | Health Scientist Administrator | Federal agency |
| Monica E. Peek, MD, MPH, MSc | University of Chicago, Section of General Internal Medicine | Ellen H. Block Professor of Health Justice | Research/academia |
| Rosemary Thomas, MPH, CHES | Penn Medicine | Director of Operations, Health Equity Advancement in Program for LGBTQ Health | Patient/consumer representative |
| Vivian L. Towe, PhD, MSc, MA | Patient-Centered Outcomes Research Institute | Senior Program Officer | Other |
| Michelle S. Williams, PhD, MSPH, MCHES | George Mason University | Associate Professor | Research/academia |
| Anonymous | - | - | 1 from a Federal agency  1 Patient/consumer representative  2 from Healthcare systems  1 from Research/academia |

Disparities: Technical Expert Panel and Key Informant Interview Questions

1. Tell us about your [organizational] work on addressing health disparities?
2. What are critical elements of successful strategies to address health disparities in prevention and health promotion?
3. What components of previously studied interventions to reduce disparities should be replicated and scaled?
4. Do you know of any other innovative or collaborative efforts on strategies to address health disparities specific to preventive or primary care?
5. Are there other funders and sponsors of work addressing health disparities specific to preventive or primary care?
6. What key financial and governance policies are needed to support interventions to mitigate health disparities?

# Online Appendix B Additional Results

**Table B-1**. Strategies to Address Social Drivers of Clinical Preventive Service Receipt

| - Partner with communities to develop implementation plans to address social needs of the community - Consider/improve the safety of the communities surrounding clinical services - Address local and state policies aimed at reducing rights, freedom, access to care for marginalized populations, and the need for supporting good social policy overall - Hold health systems and other systems (e.g., schools) accountable for strategies to improve health literacy in tandem with strategies to improve healthcare consumerism (i.e., patients as healthcare consumers) - Screen for social needs during appointments - Expand the provision of services beyond the traditional primary care settings (e.g., trustworthy and accessible community sites, homes, or mobile sites) - Deliver services through paraprofessionals (e.g., patient navigators, community health workers) by formally recognizing and reimbursing the scope of work for these professions - Ensure that the clinical workforce—from administrative staff to executive leadership—reflects the community - Extend the hours of clinical sites (evening and weekend hours) and offer home-based tests or products - Consider patients’ childcare needs - Provide culturally sensitive care teams and multilingual forms - Provide travel vouchers to appointments - Provide free or low-cost preventive services |
| --- |

**Table B-2**. Characteristics of Successful Community Partnerships

| - Infrastructure supporting local community-based organizations - Electronic platforms to facilitate linkages and partnerships - Primary care engagement and mission-driven practices - Longevity of partnerships between primary care practices and communities - Delivery sites within communities - Openness to providing care in “new” locations (e.g., COVID-19 vaccinations and testing in pharmacies) |
| --- |

**Table B-3**. Key Lessons Learned for Community Engagement

| - Understand that relationships between communities, stakeholders, and clinical partners at all levels are critical and require intentional planning, financial investments, and ongoing management support. - Before taking action, work with community members to ensure that the need you seek to address exists and is a priority for that community. Even better, work with communities proactively to identify key issues and priorities that are important to them and then work to address those priorities. - Identify local resources and strengths within the community, and leverage those resources and strengths, rather than focusing on resource limitations or challenges. - Apply a systems-level approach to share resources brought by diverse stakeholders. - Ensure diversity in teams so they are reflective of the entire community. This extends beyond race and ethnicity to include things like age, gender, geography, sociodemographic status, and education. - Work with communities to find and engage with a trusted messenger who can help navigate the discussions and actions needed to cocreate a system that meets the mutual needs of community members and the health systems in place. - Bring care delivery into the community to reduce common barriers related to access. This may include care delivery outside of primary care or other traditional healthcare offices to include places like pharmacies. The COVID-19 pandemic showed us that this is possible and beneficial to communities. - Acknowledge prior errors by the medical community and recognize that community forgiveness for those errors may be necessary. |
| --- |

1. Federal employees who participated as Stakeholder Panel members were not eligible for the honorarium. [↑](#footnote-ref-1)
2. Federal employees who participated as TEP members were not eligible for the honorarium. [↑](#footnote-ref-2)
